# Scrutinizing the immune defence inventory of *Camponotus floridanus* applying total transcriptome sequencing

**DOI:** 10.1186/s12864-015-1748-1

**Published:** 2015-07-22

**Authors:** Shishir K. Gupta, Maria Kupper, Carolin Ratzka, Heike Feldhaar, Andreas Vilcinskas, Roy Gross, Thomas Dandekar, Frank Förster

**Affiliations:** Department of Bioinformatics, Biocentre, University of Würzburg, Am Hubland, D-97074 Würzburg, Germany; Department of Microbiology, Biocentre, University of Würzburg, Am Hubland, D-97074 Würzburg, Germany; Department of Animal Ecology, University of Bayreuth, 95440 Bayreuth, Germany; Institute of Phytopathology and Applied Zoology, Justus-Liebig University of Giessen, Heinrich-Buff-Ring 26-32, 35392 Giessen, Germany; EMBL Heidelberg, BioComputing Unit, Meyerhofstraße 1, 69117 Heidelberg, Germany

**Keywords:** *Camponotus floridanus*, Carpenter ant, Transcriptome, Re-annotation, Immune system

## Abstract

**Background:**

Defence mechanisms of organisms are shaped by their lifestyle, environment and pathogen pressure. Carpenter ants are social insects which live in huge colonies comprising genetically closely related individuals in high densities within nests. This lifestyle potentially facilitates the rapid spread of pathogens between individuals. In concert with their innate immune system, social insects may apply external immune defences to manipulate the microbial community among individuals and within nests. Additionally, carpenter ants carry a mutualistic intracellular and obligate endosymbiotic bacterium, possibly maintained and regulated by the innate immune system. Thus, different selective forces could shape internal immune defences of *Camponotus floridanus*.

**Results:**

The immune gene repertoire of *C. floridanus* was investigated by re-evaluating its genome sequence combined with a full transcriptome analysis of immune challenged and control animals using Illumina sequencing. The genome was re-annotated by mapping transcriptome reads and masking repeats. A total of 978 protein sequences were characterised further by annotating functional domains, leading to a change in their original annotation regarding function and domain composition in about 8 % of all proteins. Based on homology analysis with key components of major immune pathways of insects, the *C. floridanus* immune-related genes were compared to those of *Drosophila melanogaster*, *Apis mellifera*, and other hymenoptera. This analysis revealed that overall the immune system of carpenter ants comprises many components found in these insects. In addition, several *C. floridanus* specific genes of yet unknown functions but which are strongly induced after immune challenge were discovered. In contrast to solitary insects like *Drosophila* or the hymenopteran *Nasonia vitripennis*, the number of genes encoding pattern recognition receptors specific for bacterial peptidoglycan (PGN) and a variety of known antimicrobial peptide (AMP) genes is lower in *C. floridanus*. The comparative analysis of gene expression post immune-challenge in different developmental stages of *C. floridanus* suggests a stronger induction of immune gene expression in larvae in comparison to adults.

**Conclusions:**

The comparison of the immune system of *C. floridanus* with that of other insects revealed the presence of a broad immune repertoire. However, the relatively low number of PGN recognition proteins and AMPs, the identification of *Camponotus* specific putative immune genes, and stage specific differences in immune gene regulation reflects *Camponotus* specific evolution including adaptations to its lifestyle.

**Electronic supplementary material:**

The online version of this article (doi:10.1186/s12864-015-1748-1) contains supplementary material, which is available to authorized users.

## Background

Insects are among the most successful animal life forms on Earth in terms of species richness and abundance. Like all other living organisms they are under permanent threat of infection by harmful microorganisms.

Insects are not endowed with an adaptive immune system and must rely entirely on innate immune mechanisms or externally applied immune defences [1, 2]. Invading microorganisms that break the primary passive protective barriers such as the cuticle or the peritrophic membrane in the gut encounter immediate-acting defence strategies such as phagocytic cells, phenoloxidase activity and reactive oxygen species. As a second line of defence a powerful antimicrobial immune response is mounted, mainly based on AMPs but also including serine proteases, stress factors and factors involved in opsonisation and clotting [3, 4]. Based on several structural features, the AMPs can be classified into several groups such as α-helical peptides, glycine-rich peptides, cysteine-rich peptides or proline-rich peptides [5].

Detection of microbial invaders is achieved by pattern recognition receptors (PRRs) which recognise conserved structural motifs of the microorganisms such as DAP- or Lys-containing PGN of respectively Gram-negative or Gram-positive bacteria [4]. The conserved molecular patterns of the microbes they recognise are called microbe associated molecular patterns (MAMPs) [6]. These PRRs then interact with cellular signalling systems such as Toll, IMD, Jak-Stat, and JNK pathways, which ultimately lead to the activation of an immune response [7, 8]. Early work on the *Drosophila melanogaster* immune system already revealed striking similarities of these signal transduction pathways with those of vertebrates. For instance, the identification of the *Drosophila* Toll receptor was a milestone discovery, since later it was found that related PRRs of vertebrates, the so-called Toll-like receptors, also play a dominant role in the innate immune system of vertebrates [8].

Here we characterise the immune system of the carpenter ant *C. floridanus*. These ants live in huge colonies of genetically highly related animals. The high density of closely related individuals within the nest may pose specific hygiene problems since pathogen transfer may be facilitated by the close contact of colony members. On the other hand, social insects have evolved many additional hygienic measures on the colony level which may improve health of the individuals, such as cleaning behaviours or the use of other external immune defences such as the application of antimicrobial secretions, a phenomenon termed ‘social immunity’ [2, 9, 10]. Thus, not only the canonical genes encoding AMPs or factors involved in signalling pathways but also genes encoding traits involved in external or social immune defences should be viewed as part of the immune system of a social insect [2]. For example, Le Conte and co-workers discovered several genes which might contribute to social immunity in honey bees [11]. A striking new finding was reported recently for the closely related ant *C. pennsylvanicus*, in which a Cathepsin D like protease was found to be transmitted to other ants by trophallaxis, leading to an increased infection resistance in the recipients [12]. The first genome sequence of a social insect, the honey bee, revealed an apparently low number of genes with immune-related functions as compared to solitary insects [13]. Accordingly, it was suggested that external immune defences including social immunity may have alleviated selection pressure from the canonical innate immune factors as internal and external immune defences may trade off against each other. However, the recent honey bee genome upgrade identified about 5000 more protein encoding genes than previously reported, which need to be analysed carefully in the future [14]. In addition, the genome sequences of several other hymenopteran and dipteran species reveal that dipterans appear to have an unusually high number of immune genes and that a social lifestyle may not directly correlate with this number [15].

A specific feature of *C. floridanus* is its obligate interaction with an intracellular mutualistic y-Proteobacterium, *Blochmannia floridanus*, which resides in midgut cells and in the ovaries and supplements nutrients to its host [16]. The host recognises the endosymbiont as non-self and the immune system can therefore play a role in maintenance and regulation of the chronic infection by the endosymbiont [17], while on the other side it has to defeat pathogenic microorganisms, thus possibly requiring specific adaptations of its immune system [18]. For instance, previous work revealed a localised down-modulation of the immune response that is restricted to the midgut tissue of the ants and correlates with massive replication of the endosymbiont in this tissue [18].

To get insight into the immune system of the carpenter ant and to unravel possible specific adaptations to its lifestyle as a social insect living in an obligate mutualistic interaction with a bacterial endosymbiont, we re-evaluated the data available from the recently published genomic sequence of *C. floridanus* [19] and extended this dataset with genome-wide transcriptome data generated from animals with or without previous immune challenge. To identify and functionally annotate the key players of the *C. floridanus* immune response and to determine the interactions among these players, we performed a detailed analysis including sequence and domain analysis and pathway annotation (including signalogs [20]). Transferring annotation of proteins at a domain level allows more accurate functional inference [21] and is useful for predicting the function of multi-domain proteins [22] and novel domain combinations that possibly give rise to new protein functions [23]. Furthermore, we compared the *C. floridanus* immune genes and pathways to other recently sequenced ant genomes including *Acromyrmex echinatior*, *Atta cephalotes*, *Cerapachys biroi*, *Harpegnathos saltator*, *Linipithema humile*, *Pogonomyrmex barbatus*, *Solenopsis invicta* [19, 24–29]. We used clusters of orthologous genes and protein families (e.g. according to PFAM) for many of the above-mentioned comparisons and thus the comparisons based on these entities are quite broad. Nevertheless, regarding the number of complete genomes available there are limitations for drawing general conclusions. Moreover, other hymenoptera such as the honey bee *A. mellifera* [13], the solitary parasitic wasp *Nasonia vitripennis* [30], and, as a model organism, *D. melanogaster* [31], served to investigate possible differences in the immune system of endosymbiont-bearing, eu-social and solitary insects.

## Results and discussion

### *C. floridanus* genome structure and re-annotation

To establish the immune repertoire of *C. floridanus* the full transcriptome was analysed by Illumina sequencing, comparing challenged and unchallenged animals (pools of whole larvae L2 and workers W2 for each sample). Sequencing resulted in 125,873,897 reads for immune-challenged and 118,142,837 reads for untreated animals. Sequence statistics of Illumina sequencing are listed in Table 1. The first *C. floridanus* genome annotation (v3.3) contains 17,064 protein-coding genes [19]. The updated version of the previous annotation is labelled as cflo_OGSv3.3 (available at http://hymenopteragenome.org/camponotus/). Compared with the previously published annotation of the *C. floridanus* genome, the optimised Augustus software with species-specific parameters applied here predicted 15,631 protein-coding genes based on data from *C. floridanus* expressed sequence tags (ESTs) and the large-scale Illumina sequencing data (raw reads and assembled reads) presented here. Our revised annotation counts fewer genes and an increase in the number of multi-exon genes as compared to the published version v3.3. The accuracy of annotation for both was improved using the latest version of the gene prediction tool Augustus v2.7. Furthermore, over-prediction (false positives) of single exon genes was reduced by this software. The improved quality of the annotation was further validated as 14,956 (81.41 %) predicted transcripts could now be supported by extrinsic evidence such as introns and exons hints generated by sources such as Illumina sequence data and ESTs. The new annotation predicts a total of 18,369 proteins as compared to 17,064 proteins in the previous version v3.3. We found 1928 genes with two or more alternative transcripts. The optimised parameters with 80.8 % exon level accuracy allowed the improvement of annotation of the *C. floridanus* genome (http://bioinf.uni-greifswald.de/augustus/binaries/species/), e.g. with regard to splicing events. The prediction accuracy of Augustus with the new optimised parameters is tabulated in Additional file 1: Table S1. The optimised parameters are provided as part of the latest Augustus package (http://bioinf.uni-greifswald.de/augustus/submission/). Bonasio and co-workers (2011) detected 7583 alternative splicing events in 2538 genes (cflo_OGSv3.3) overall, while our analysis revealed 1928 genes affected by alternative splicing events coding for 4666 alternative transcripts (Additional file 2: Table S2). However, the OGSv3.3 data available for *C. floridanus* contain 17,064 transcripts and 17,064 proteins without distinction between alternative isoforms and thus cannot be used for further analysis. The new data reported here can be accessed at our web repository (http://camponotus.bioapps.biozentrum.uni-wuerzburg.de) and distinguish the alternative splicing products of the genes with the suffix in the accession number as t1, t2, t3 etc. The exact distribution of alternative transcripts over the annotated 15,631 genes is listed in Additional file 1: Table S1.

**Table 1:** Quantitative overview on the transcriptome sequencing data

(A) Illumina sequencing – quantitative overview			
		Immune challenged	Non-immune challenged
Sequencing		Paired end 2 x 50 bp	
Total number of reads		125,873,897	118,142,837
Median insert length (bp)		176 bp	163 bp
Overall mapping rate		87.4 %	88.6 %
Mapping to *Camponotus* genome		99.45 %	99.37 %
Mapping to *Blochmannia* genome		0.55 %	0.63 %
(B) Repeats distribution			
	Number of elements	Length occupied	Percentage of sequence
LTR elements	662	445,584 bp	0.19 %
DNA elements	163	44,183 bp	0.02 %
Unclassified	41,722	14,401,396 bp	6.13 %
Total interspersed repeats	-	14,891,163 bp	6.34 %
Small RNA	26	13,938 bp	0.01 %
Satellites	50	9914 bp	0.00 %
Simple repeats	67,608	3,996,593 bp	1.70 %
Low complexity	284,104	15,360,412 bp	6.54 %

We compared the improved genome annotation of *C. floridanus* to published data on other genomes, showing that *C. floridanus* has fewer repetitive elements (15.05 %) than *D. melanogaster* (27.38 %) or *N. vitripennis* (24.31 %), but more than *A. mellifera* (6.86 %). Using the major database of repetitive elements, Repbase [32], we list 12 specific repetitive elements for the *C. floridanus* genome and we give a summary of these elements in Table 1. Based on a *de novo* repeat library constructed with the RepeatModeler programme we detected 62 repetitive elements in the *C. floridanus* genome. With the assembled repeat library, 14.57 % of the *C. floridanus* genome was identified to contain repetitive sequences, consisting of 6.34 % of interspersed repeat elements, 1.70 % of simple repeats, 6.54 % low complexity stretches, and 0.01 % of small RNAs and satellites (Table 1).

### Functional annotation and classification

**Fig. 1:**
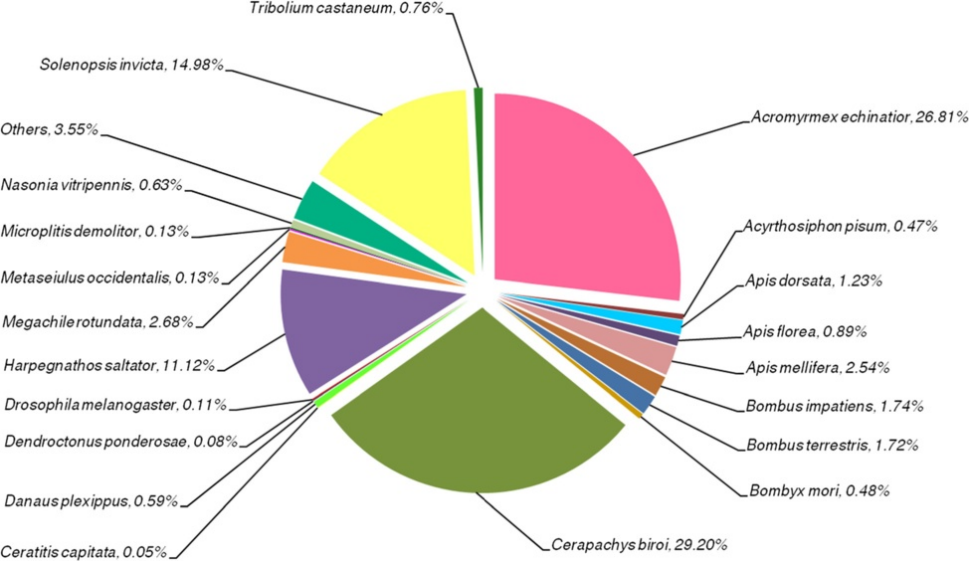
Distribution of closest related sequences to *C. floridanus* proteins. All proteins within the NCBI nr database (without *C. floridanus*) were analysed using the sequence comparison tool BLAST [118] with an *e*-value cut-off of 1e-5. The best sequence-similar hit of each re-annotated *C. floridanus* sequence was used for the analysis

**Fig. 2:**
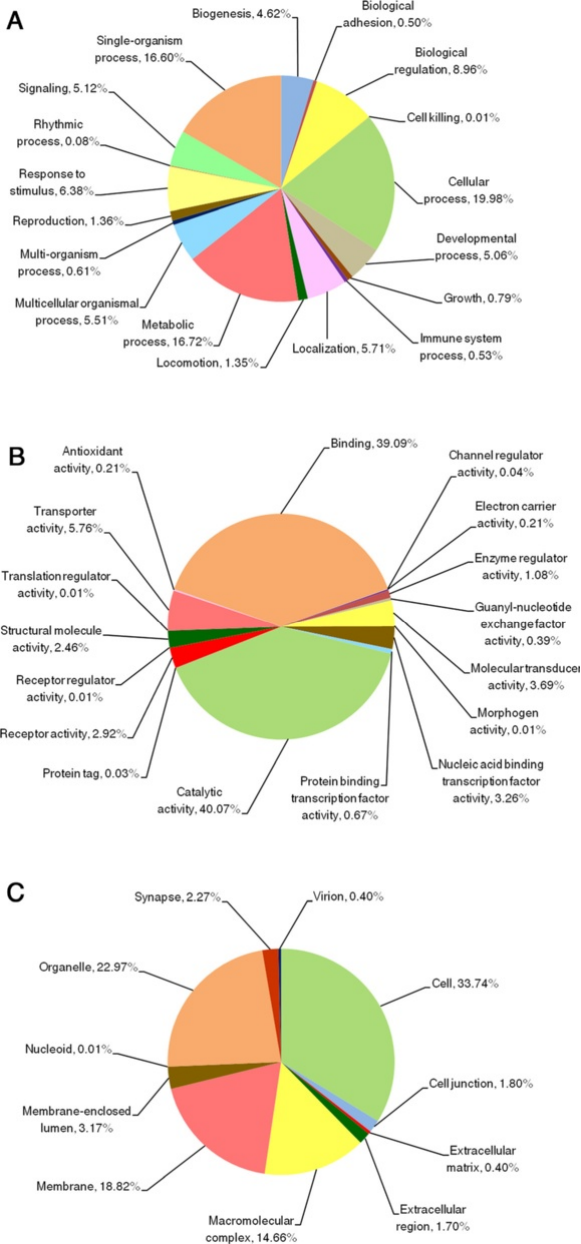
Distribution of functions in *C. floridanus* proteins. Categorisation of 8490 proteins *of C. floridanus* in GO terms (level two) for **a** biological process, **b** molecular function, and **c** cellular component with a filter score *e*-value cut-off of 1e-5

In comparison to the previous *C. floridanus* genome annotation (v3.3) we found 978 more proteins with functional domains (Additional file 3: Figure S1; new annotation Cflo-New). These protein sequences were further analysed with regards to their function. The total number of additional proteins identified was higher but not all of them contained functional domains. We further compared the changes in level-2 GO annotations in all three GO categories, i.e. biological process, molecular function and cellular component. As expected, based on best hits of sequence-based similarity searches we found that the *C. floridanus* sequences are generally most closely related to recently sequenced ant species (Fig. 1). Furthermore, we examined their overall functions. Among the 18,369 proteins, the software Blast2GO assigned level 2 GO terms to 8490 proteins and stressed important functions (Fig. 2). Regarding the term “biological process” (Fig. 2a), various “cellular processes” (green) were most abundant (19.98 %), regarding “molecular function” (Fig. 2b), enzymes presented the highest fraction among the proteins (“catalytic activity”, 40.07 %, green), directly followed by “binding” with 39 %. Regarding “cellular compartment” the subcategory “Cell” with 33.74 % was found to be most abundant (Fig. 2c). However, consider that two thirds of the proteins are located in membranes, organelles or part of macromolecular complexes (Fig. 2 shows in detail all other functional categories). In total 7143 proteins of Cflo(v3.3) could be annotated by Blast2GO. The proportion of Cflo(v3.3) proteins annotated previously falling into each functional category is shown in Additional file 4: Figure S2. Comparing our functional annotation with Cflo(v3.3) in terms of GO classification, we note an increase of 18 to 23 % regarding successful subcategory assignments covering all three major GO categories (13,354 terms in comparison to 10,819 terms in Cflo(v3.3) for “biological process”; 6088 terms versus 5141 in “molecular function”, and 5441 terms versus 4568 terms respectively for the GO category “cellular component”; Additional file 5: Table S3). In conclusion, by refining the accuracy of prediction regarding single and multiple transcript genes and evidence for their expression the transcriptome sequencing improved the annotation of the *C. floridanus* proteome, the identification of repetitive elements as well as alternative splicing predictions.

### Comparison of the immune gene repertoire of *C.floridanus* with different insect species

**Fig. 3:**
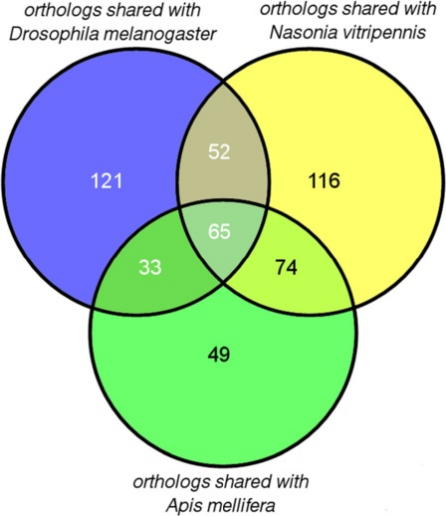
Immune gene repertoire of C. *floridanus* shared with different insect species. The Venn diagram shows the number of immune protein orthologs (including alternative splice isoforms) of *C. floridanus* shared with *D. melanogaster*, *N. vitripennis* and *A. mellifera*

**Fig. 4:**
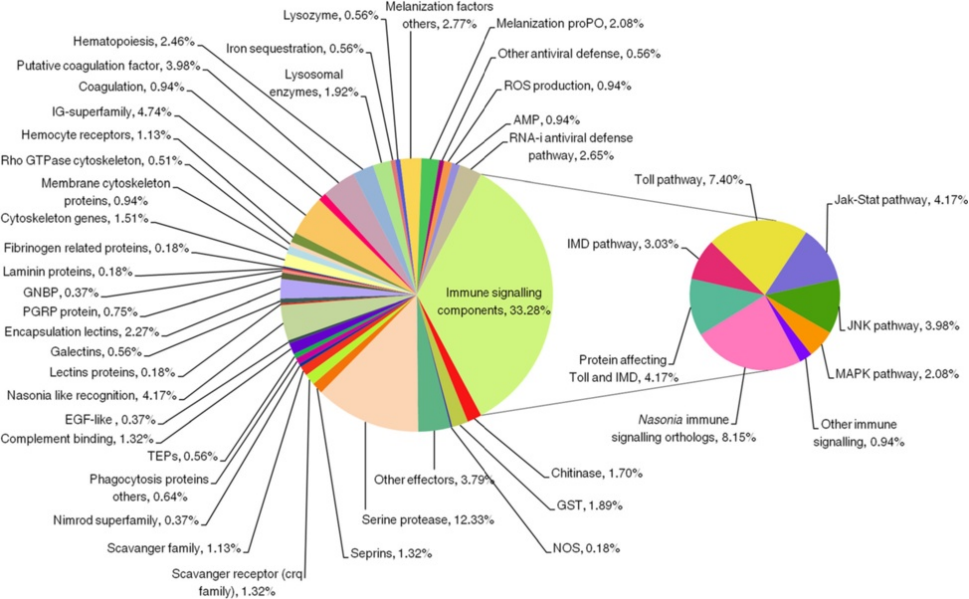
Functions of immune-related genes of *C. floridanus*. The pie chart shows the percentage of immune genes (474 genes in total) in each functional sub-category relative to the entire set of immune genes. The category ’immune signalling components’ is further divided into different pathways

We present the *C. floridanus* immunome using sequence-based protein orthology with the previously published data of *D. melanogaster*, *A. mellifera* and *N. vitripennis* assuming their functions and modes of action are conserved (Fig. 3). *C. floridanus* shares 307 orthologs with *N. vitripennis*, 271 orthologs with *D. melanogaster*, and 221 with *A. mellifera*. All four species share 65 immune protein orthologs which mostly comprise proteins involved in core immune signalling pathways, PRRs or serine proteases (Additional file 6: Table S4). Overall, in accordance with previous findings [33] signalling pathways seem to be more conserved in comparison to effector molecules that show higher levels of taxon specificity. We classified the 474 immune related genes of *C. floridanus* identified here into several categories including microbial recognition, signalling pathways (Toll, Jak-Stat, IMD and JNK), AMPs, phagocytosis, melanisation, encapsulation, cytoskeleton immune proteins, antiviral defence, coagulation, haematopoiesis and other immune responses (Fig. 4). In the Additional file 7: Table S5 the accession numbers and annotations of all categorised immune proteins of *C. floridanus* are listed.

### Signal transduction via major immune signalling pathways

Recognition of a pathogen is the first step in promoting an efficient immune response. Therefore, pattern recognition receptors (PRRs) recognise the so-called MAMPs such as bacterial PGN or fungal beta-1,3-glucans [4, 6]. Upon binding to microbial components they trigger the activation of signal transduction systems either directly or after a series of proteolytic events mediated by serine proteases, ultimately resulting in the activation of antimicrobial defence mechanisms including the expression of AMPs [4]. Due to the in-depth knowledge and functional assays of its major immune signalling pathways [34, 35] we used *D. melanogaster* as a template to identify components of the Toll, IMD, Jak-Stat, and JNK signal transduction pathways in *C. floridanus*. The reconstructed immune related signalling repertoire of *C. floridanus* is highly conserved and largely similar to the signalling components identified by experiments mainly conducted with *Drosophila* [36].

#### Toll signalling pathway

**Fig. 5:**
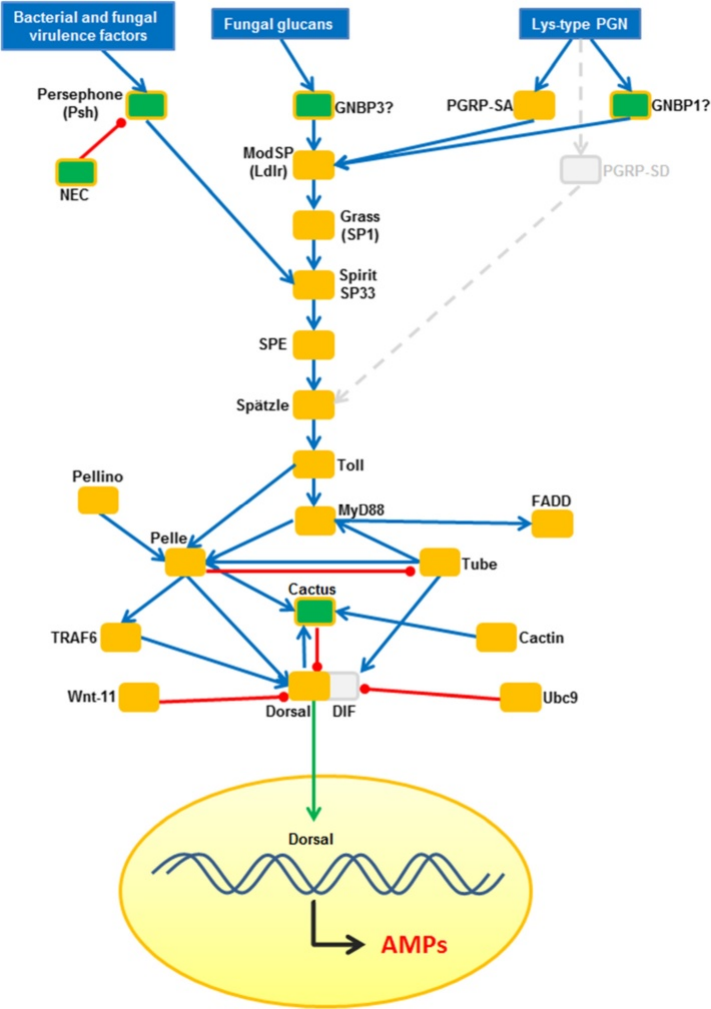
The Toll signalling pathway of *C. floridanus*. All identified signalling components are mapped on the comprehensive immune network of *D. melanogaster*. The names of the factors correspond to the *Drosophila* designations. Connectivity among nodes is based either on positive attribute (blue arrow) or negative attribute (red arrow). Missing components are shown in grey colour. Nuclear translocation is shown by a green arrow. Factors significantly upregulated on the transcriptional level upon immune-challenge are shown by green boxes

The Toll pathway of insects is mainly activated by fungal pathogens and Gram-positive bacteria. The Toll pathway not only regulates the antimicrobial response but is also required for proper haemocyte proliferation [4, 37]. Therefore, Toll activation leads to a coordinated immune response that comprises both cellular and humoral immunity [38]. The Toll signalling pathway was found to be highly conserved in terms of the presence of homologs in *C. floridanus* (Fig. 5). Recognition of Lys-type PGN characteristic for most Gram-positive bacteria or of fungal beta-1,3-glucans by specific PRRs leads to the activation of proteolytic cascades which finally activate the Toll-dependent signalling cascade. In *C. floridanus* three PRRs likely feeding into the Toll pathway are found: a PGRP-SA (Cflo_N_g8526t1) which according to sequence homology probably recognises Lys-type PGN, and two proteins annotated as beta-1,3-glucan binding proteins (Cflo_N_g15215t1 and Cflo_N_g5742t1) with high homologies to both GNBP1 and GNBP3 of *D. melanogaster*. GNBP1 is known to perceive Lys-type PGN, while GNBP3 recognises fungal cell wall components [39]. The homology data of the *C. floridanus* proteins do not allow a clear identification of the signal sensed by the two GNBPs. However, the previous expression data acquired post immune challenge of *C. floridanus* with Gram-negative or Gram-positive bacteria revealed a long-lasting up-regulation of the gene encoding one of the GNBPs (EFN66519.1; Cflo_N_g5742t1) only after infection with Gram-positive bacteria [17]. This suggests that this *Camponotus* protein may be able to recognise Lys-type PGN and thus may be a functional homolog of GNBP1 of *D. melanogaster* (Fig. 5). In addition, *C. floridanus* encodes a homolog of the protease Persephone which was previously shown to be involved in the detection of danger signals indicative for infection with Gram-positive bacteria and fungi [40]. In *D. melanogaster* the Toll pathway is triggered upon microbially induced proteolytic cleavage of the circulating cytokine-like ligand molecule Spätzle that binds to the Toll receptor, thus finally leading to the nuclear translocation of the NF-κB-like transcription factors Dorsal and DIF (Dorsal-related immunity factor). A single gene encoding Dorsal is present in *C. floridanus*, but similar to *A.mellifera* no ortholog of DIF was found. This is in agreement with the recent suggestion that DIF belongs to a highly derived branch possibly found only in brachyceran flies [13]. Therefore, in *C. floridanus* Dorsal appears to be the unique transcription factor required for induction of AMPs during the Toll mediated immune response.

#### IMD and JNK signalling pathways

**Fig. 6:**
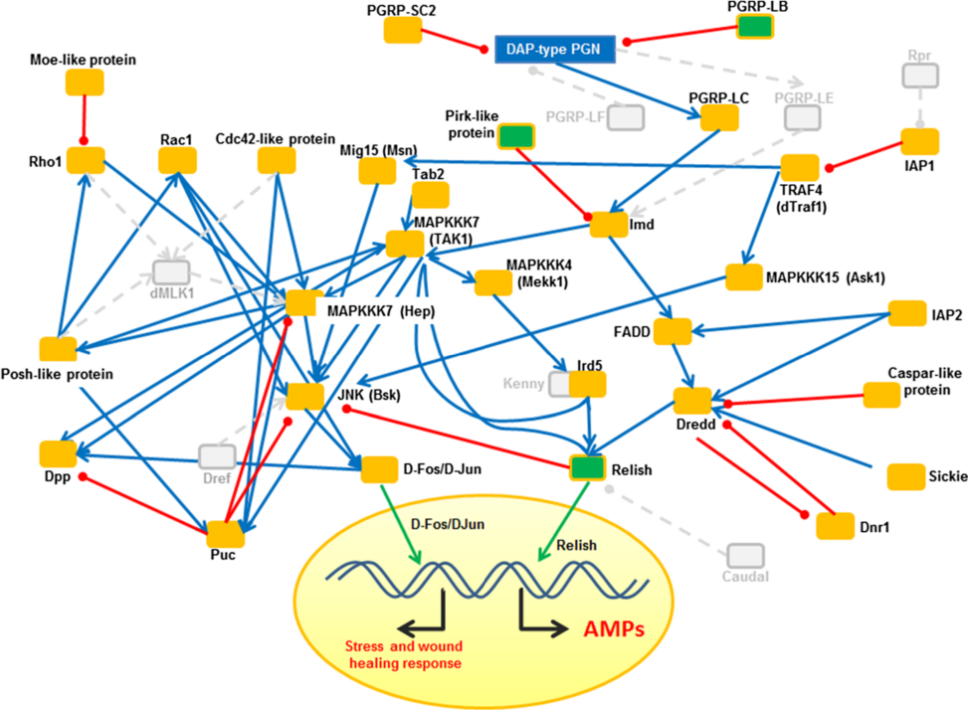
The Imd and JNK pathways of *C. floridanus*. All identified signalling components are mapped on the comprehensive immune network of *D. melanogaster*. The names of the factors correspond to the *Drosophila* designations. Connectivity among nodes is based either on positive attribute (blue arrow) or negative attribute (red arrow). Missing components are shown in grey colour. Nuclear translocation is shown by a green arrow. Factors significantly upregulated on the transcriptional level upon immune-challenge are shown by green boxes

The activation of the IMD pathway of insects is triggered after infection predominantly by Gram-negative bacteria and is also involved in the induction of expression of AMPs [4, 41]. *C. floridanus* harbours the Gram-negative obligate intracellular endosymbiont *B. floridanus* and it was suggested that the IMD pathway may also contribute to control and tolerance of the endosymbiont [17, 18]. The present data show that most components of the IMD pathway are present in *C. floridanus* (Fig. 6). However, in *C. floridanus* DAP-type PGN mainly characteristic for Gram-negative bacteria is recognised via a single signal-transducing PRR, originally annotated as PGRP-LE, while the organism appeared to lack PGRP-LC-like receptors. The careful re-evaluation of the genomic data revealed, however, that this protein has a much longer N-terminal sequence and comprises a transmembrane domain at sequence position 264 to 287. In fact, a cluster analysis revealed that the protein is more related to PGRP-LC sequences of *D. melanogaster* and *A. mellifera* than to PGRP-LE of *D. melanogaster*. Thus, the *C. floridanus* protein is likely to be a PGRP-LC homolog (Cflo_N_g10272t1) and appears to be the only receptor for DAP-type PGN that activates the IMD pathway. In addition, two PGN recognition proteins with regulatory function (PGRP-SC and PGRP-LB) were previously found [18]. Due to the presence of an amidase domain both of them may down-modulate the signal transduction pathways by cleavage of PGN [6, 42]. In fact, PGRP-LB was recently found to be implicated in the tolerance towards the obligate intracellular endosymbiont *B. floridanus* in the midgut tissue during pupation of the animals. PRGP-LB is highly up-regulated only in the midgut tissue and not in other parts of the pupa body cavity, coinciding with a massive multiplication of the endosymbiont and a reduction of the immune competence in this tissue [18].

A major difference with regard to *D. melanogaster* in the cytosolic *C. floridanus* IMD signalling cascade concerns the so-called IKK complex which activates the NF-κB-like transcription factor Relish by phosphorylation [43]. In *Drosophila* the IKK complex is composed of the enzymatically active Ird5 subunit and the regulatory subunit Kenny. However, in *C. floridanus* the regulatory subunit is missing. It has been published that a Kenny mutant of *D. melanogaster* is highly susceptible to bacterial infections [44]. Iterative sequence analyses also verified the lack of the Kenny subunit in *A. mellifera*, *N. vitripennis* and other ant species, suggesting a common character of the IKK complex in hymenoptera. Whether the lack of the Kenny subunit may reflect a reduction in the immune potential of hymenoptera or whether so far unknown factors may be involved in building a functional IKK complex is not yet known.

The IMD pathway also leads to TAK1 (transforming growth factor β-activated kinase 1) mediated activation of the JNK signalling cascade [45]. JNK signalling contributes to regulation of many developmental processes [46], wound healing [47], activation of stress-protective proteins [48], inflammatory [49] and cellular immune responses [50]. The data reported here indicate that the JNK pathway in *C. floridanus* is quite similar to the pathway in *D. melanogaster*, since most of the core components of the JNK pathway of *D. melanogaster* have homologs in *C. floridanus* (Fig. 6).

#### Additional receptor proteins and Jak-Stat signalling pathway

**Fig. 7:**
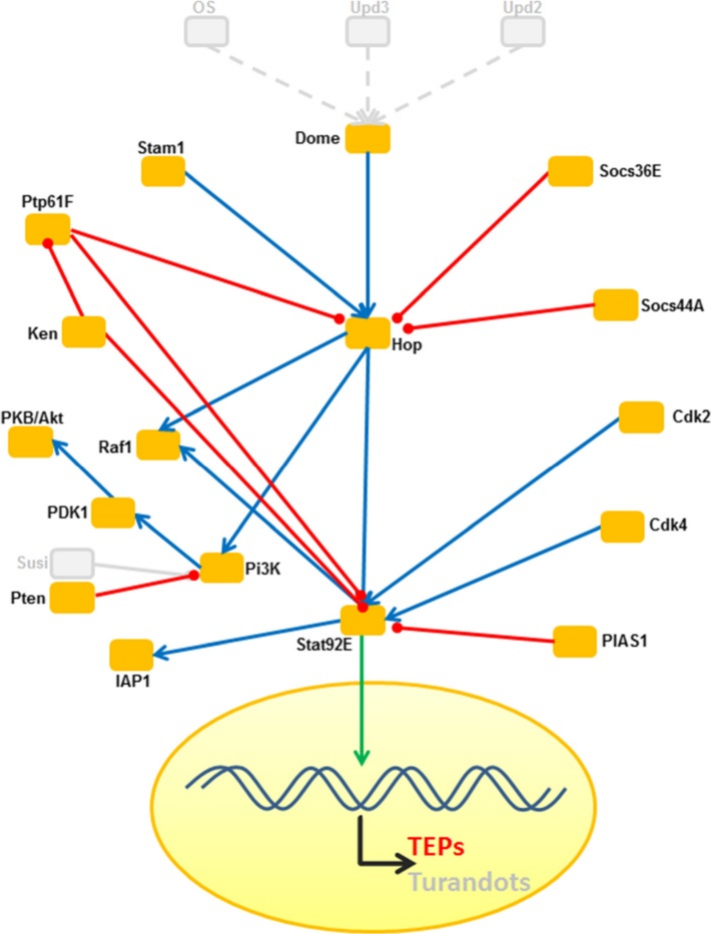
The Jak-Stat pathway of *C. floridanus*. All identified signalling components are mapped on the comprehensive immune network of *D. melanogaster*. The names of the factors correspond to the *Drosophila* designations. Connectivity among nodes is based either on positive attribute (blue arrow) or negative attribute (red arrow). Missing components are shown in grey colour. Nuclear translocation is shown by a green arrow. Factors significantly upregulated on the transcriptional level upon immune-challenge are shown by green boxes

Besides the above mentioned PRRs, we also identified proteins known to be involved in pathogen recognition and/or promotion of phagocytosis including scavenger receptors, croquemort family members, nimrod and draper orthologs, vitellogenin, galectins, c-type lectins, brain angiogenesis inhibitor 1 (BAI1), fibrinogen-related protein, down syndrome cell adhesion molecular (Dscam) and thioester containing proteins (TEPs). The latter are known to play a role in the Jak-Stat pathway, which contributes to stem cell regulation in the intestine of *Drosophila* and thus to midgut homeostasis [51, 52]. In *C. floridanus* all core components of the Jak-Stat pathway are present (Fig. 7) except extracellular ligand proteins identified in *Drosophila,* which activate the pathway and which are also not found in other insects, including the honey bee [13]. While TEPs and Turandot proteins are among the downstream effectors of the Jak-Stat pathway in *D. melanogaster* [53, 54], similar to *A. mellifera* no homologs of Turandot proteins were identified by BlastP searches in *C. floridanus,* but several TEPs were found. TEPs may play a role in insect immunity by promoting phagocytosis of bacteria [53, 55]. Most TEPs share the common CGEQ motif defining the thioester site, which allows the formation of a covalent bond to microbial surfaces [55]. However, several TEPs in insects lack the thioester motif, but these TEPs may act as adaptors for the initiation of the membrane attack complex as is found in vertebrate complement factors [53]. Sequence analyses revealed the presence of three TEP genes (TEP1: Cflo_N_g4492t1, TEP2: Cflo_N_g7345t1, and TEP3: Cflo_N_g9745t1) in *C. floridanus*, with TEP1 and TEP2 containing the CGEQ motif in the deduced amino acid sequences. For the gene encoding TEP3, two alternative transcripts were found (Cflo_N_g9745t1 and Cflo_N_g9745t2). Interestingly, only the CGEQ motif containing TEP1 of *C. floridanus* is up-regulated upon immune challenge (see below).

### Antimicrobial peptides of *C. floridanus* and other hymenoptera

The previous analysis of the *C. floridanus* genome revealed a relatively low number of known AMPs including two defensins, a hymenoptaecin, a tachystatin-like and a crustin-like peptide [56–58]. These peptides are also encoded by most of the other ants [58], however, the distribution pattern of AMPs is in general quite complex (Table 2). For example, several of the ants including *C. floridanus* lack a gene encoding abaecin, while other ants encode this AMP. Thus, similar to the previously described gain, loss and duplication of *defensin* genes [56], there is quite an extensive variability in the presence and number of antimicrobial peptide genes in the ant genomes (Table 2). Much alike in the honeybee, the number of predicted or confirmed AMPs appears to be relatively low in ants as compared to the solitary wasp *N. vitripennis* for which 44 AMPs were described [57]. The apparently low number of AMPs may be compensated by the astonishing gene structure of hymenoptaecin which in the ants is encoded as a huge precursor protein with several repeated hymenoptaecin domains. Proteolytic maturation of this precursor protein leads to a massive amplification of the immune response [56]. In addition, the *hymenoptaecin* gene is among the most strongly induced genes after immune challenge (see below). The apparently quite low number of AMPs and of PGN recognising PRRs as described above may relate to the social lifestyle of ants and bees as compared to the solitary and parasitic lifestyle of the wasp *N. vitripennis*, since social life might allow hygienic measures on the colony level [9]. In addition, ants produce a range of antimicrobial secretions that may be used to reduce pathogen pressure externally before an infection of the body occurs. As a consequence these external immune defence strategies may trade off against internal immune defences and may result in a reduction in the number of effector molecules [2, 10].

**Table 2:** Antimicrobial peptides of *C. floridanus* and other hymenoptera

AMPs	*C. floridanus*	*H. saltator*	*L. humile*	*P. barbatus*	*A. cephalotes*	*S. invicta*	*A. echinatior*	*C. biroi*	*A. mellifera*	*N. vitripennis*
Hymenoptaecin^1)^	1	2	1	1	1	1	3	1	1	2
Defensin	2	2	1	5	1	2	1	1	3	5
Tachystatin-like	2	2	3	2	1	3	2	3	1	3
Crustin-like^2)^	1	1	1	1	1	1	1	-	-	1
Abaecin	-	1	-	1	1	-	1	1	1	-
Melittin	-	-	-	-	-	-	-	-	1	-
Apisimin	-	-	-	-	-	-	-	-	1	-
Apidaecin	-	-	-	-	-	-	-	-	5	-
Navitripenicin	-	-	-	-	-	-	-	-	-	4
Nasonin^3)^	-	-	-	-	-	-	-	-	-	14
Nabaecin^4)^	-	-	-	-	-	-	-		-	4
Glynavicin^3)^	-	-	-	-	-	-	-	-	-	7
Hisnavicin	-	-	-	-	-	-	-	-	-	5
Nahelixin	-	-	-	-	-	-	-	-	-	1

^1)^Please note that here the number of genes present in the various species is indicated. In the ants the hymenoptaecin genes encode huge multipeptide precursor proteins which may give rise to several mature peptides (7 in the case of *C. floridanus*)

^2)^Adopted from Zhang and Zhu [58]

^3)^Adopted from Sackton et al. [33]

^4)^Nabaecin of *N. vitripennis* is considered as a member of the abaecin family. However, nabaecins belong to different orthologous cluster; therefore we separated nabaecins and abaecins

### Prophenoloxidase, serine proteases and serpins

Several immune defence reactions in insects such as phagocytosis, melanisation and nodulation depend on phenoloxidase (PO) activity [59–61]. During the melanisation process toxic intermediates such as reactive oxygen species (ROS) may kill microbial invaders directly. Since phenoloxidase activity can also harm insect cells the enzyme is synthesised as an inactive precursor (Pro-PO). Pro-PO activation involves microbial recognition by PRRs and proteolytic cascades involving terminal serine proteases that finally cleave Pro-PO to its active form [60, 62]. Serpins negatively control the activity of PO and help to avoid overshooting melanisation and dangerous ROS production [63]. Phenoloxidases are related to arylphorins, hemocyanins and hexamerins [64]. Using query sequences from four insect species, BlastP searches resulted in eight significant hits. The first hit corresponds to the *C. floridanus* prophenoloxidase (Cflo_N_g1918t1), while the other hits are distributed among hemocyanin, arylphorin and hexamerin sequences. Thus, a single *prophenoloxidase* gene appears to be present in the *C. floridanus* genome.

In addition, we annotated 34 serine proteases and ten serine protease inhibitors in *C. floridanus* (Additional file 8: Table S6) using the AutoFACT v3.4 tool for functional annotation of gene models [65]. Among these are five putative immune related serine proteases and four serine protease inhibitors including one serpin that showed differential expression profiles after immune challenge (see below).

### Chitinases, glutathione-S-transferases and nitric oxide synthase (NOS)

Enzymes with chitinase activity play an important role in the immune defence of insects by catalysing the breakdown of chitin, a linear polymer found in fungal pathogen cell walls consisting of β-1-4 linked N-acetylglucosamine [66]. Four conserved motifs (KXXXXXGGW, FDGXDLDWEYP, MXYDXXG and GXXXWXXDXD, where X is a non-specified amino acid) have been reported in catalytic domains of insect chitinases [67, 68]. Five prototypic chitinases from different insect species served as a standard for detection [68–71]. In *C. floridanus* we found putative 13 chitinases containing a variable number of the four conserved sequence motifs (Additional file 9: Table S7). Only two predicted proteins are endowed with all four chitinase signature motifs, while in five sequences no such motif was detected. Overall, there is not much variation in the number of *chitinase* genes encoded by the different insect species compared here (Additional file 10: Table S8).

Glutathione S-transferases (GSTs) comprise a diverse family of dimeric enzymes that have attracted attention in insects because of their involvement in the defence towards insecticides [72]. Cytosolic GSTs in insects have been assigned to six classes including delta, epsilon, omega, sigma, theta and zeta [73, 74], and among them the delta and epsilon classes represent over 65 % of the total GST expansions. A recent phylogenetic study of insect GSTs suggested the evolution of the epsilon class from the delta class [66]. We predicted several *C. floridanus* GSTs that were classified into different classes on the basis of their sequence similarities and phylogenetic relationships with other insect species. Based on these approaches, 9 out of 10 identified *C. floridanus* GSTs were assigned to five different classes, including three in omega, three in sigma, one in each of delta, theta and zeta and one unclassified GST (Additional file 11: Figure S3). The absence of epsilon class GSTs in *C. floridanus* is in line with their absence in other Hymenoptera [75]. With the exception of *D. melanogaster* encoding 20 GSTs, there are only minor differences in the number of GSTs encoded by the other insects (between eight and eleven GSTs) (Additional file 10: Table S8).

NOS belongs to the family of enzymes which form nitric oxide (NO) from L-arginine and makes important contributions to the IMD pathway in activation of Relish, since NOS activity is required for a robust innate immune response to Gram-negative bacteria in *Drosophila* [76, 77]. In *C. floridanus* only a single gene (Cflo_N_g5430t1) codes for a protein that matched all criteria of a NOS. With the exception of *A. cephalotes* and *N. vitripennis* each encoding two NOS, the other ants and *A. mellifera* code for a single NOS (Additional file 10: Table S8).

### Identification of genes differentially expressed (DEGs) after immune challenge

**Fig. 8:**
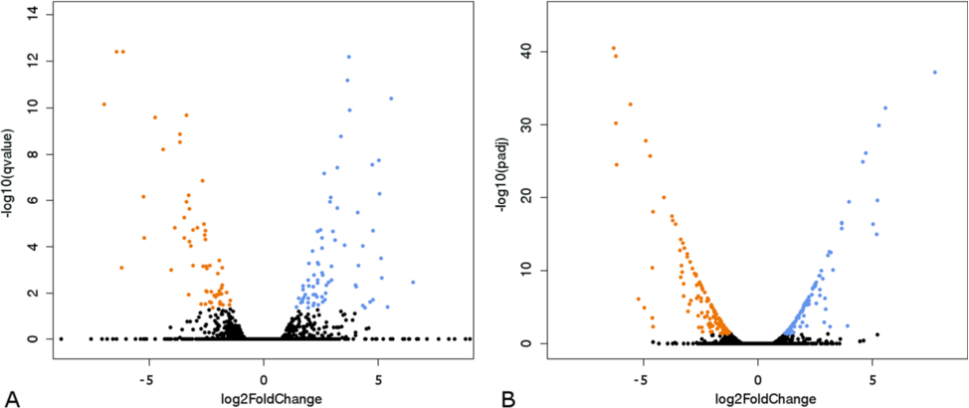
Gene expression changes after immune challenge. Volcano plots show the statistical significance of the difference in expression observed (*p*-value from a t-test, *q*-value in case of Cuffdiff and adjusted *p*-value in case of DEseq; log10 scale). The x-axis indicates the differential expression profiles, plotting the fold-induction ratios in a log-2 scale during immune challenge. The list of significantly differentially expressed protein coding genes can be found in Table S9 (Additional file 12). **a** Volcano plot from Cuffdiff data. Up-regulated genes (*q*-value < 0.05 and log2FoldChange ≥1) are shown as blue dots and the down-regulated genes (*q*-value < 0.05 and log2FoldChange ≤ −1) are shown as orange dots. The top three protein coding genes most up-regulated are Cflo_N_g6748 (hypothetical), Cflo_N_g2827 (voltage-dependent calcium channel type A subunit alpha-1) and Cflo_N_g12631 (serine proteinase Stubble). The three most down-regulated genes include Cflo_N_g2215 (putative chitin binding peritrophin-a domain containing), Cflo_N_g1319 (serine protease inhibitor 3) and Cflo_N_g4308 (lipase member H). **b** Volcano plot from DEseq data. Up-regulated genes (adjusted *p*-value < 0.05 and log2FoldChange ≥1) are shown by blue dots and the down-regulated genes (adjusted *p*-value < 0.05 and log2FoldChange ≤ −1) are shown by orange dots. The top three protein coding genes most up-regulated are Cflo_N_g6748 (hypothetical), Cflo_N_g5222 (hypothetical) and Cflo_N_g531 (aminopeptidase N). The three top down-regulated genes include Cflo_N_g2215 (putative chitin binding peritrophin-a domain containing), Cflo_N_g1319 (serine protease inhibitor 3) and Cflo_N_g907 (chymotrypsin-1)

In the previous sections immune genes were identified mainly based on their similarity to already described immune genes of other insects. To further strengthen this analysis and to identify additional factors possibly involved in immune reactions, the transcriptome analysis presented here was performed with immune challenged and untreated animals (pooling larvae and adult workers). Genes found to be regulated by immune challenge are likely to be involved in immune functions, although it is known that there are many regulatory overlaps with stress responses other than immune challenge [78]. We used two different programmes to evaluate differentially expressed genes between these samples. The combination of the results from Cuffdiff (*q*-value < 0.05) and DESeq (*p*-value adjusted < 0.05) allowed the identification of 257 transcripts, which were significantly differentially expressed in response to bacterial challenge. To show at the same time amount of change and statistical significance, Volcano plots summarise the results (Fig. 8). Table S9 (Additional file 12) lists all genes that were up- or down-regulated by at least a factor of 2 including their annotation and log fold change expression value. Among the differentially expressed transcripts, ~20 % of transcripts were identified to code for known immune related proteins or proteins described to be differentially regulated after immune challenge in other insects. The percentage of immune-related genes in the genome is quite low. According to the functional categories shown in Fig. 2 only 0.53 % of genes are related to immune processes. Even when taking into account the limits of the accuracy of functional categories, all other signalling processes together encompass only 5.12 % of all genes. Hence, there is enrichment for immune related genes regarding the much higher percentage within all differentially regulated genes. Genes up-regulated after immune challenge encode well known immune related genes including those that encode PRRs and serine proteases (e.g. Snake and Stubble-like), proteins involved in signalling and transcription (e.g. nuclear factor NF-kappa-B p110 subunit (Relish), NF-kappa-B inhibitor (Cactus), as well as stress-related proteins such as cytochromes P450.

**Fig. 9:**
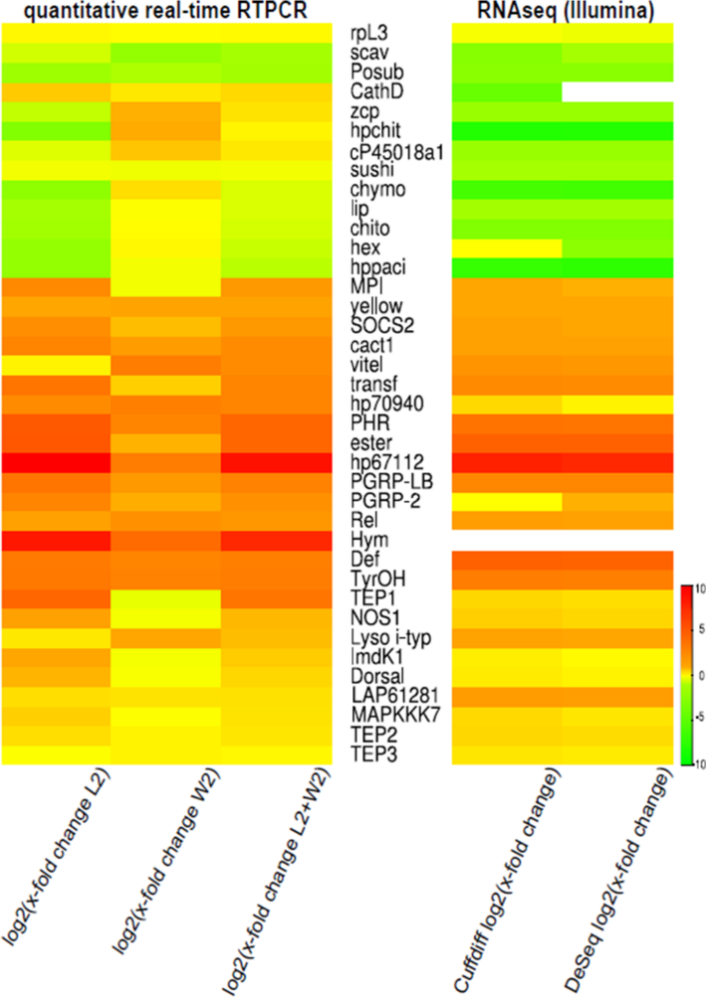
Comparison of expression of 37 selected genes based on the analysis of the Illumina sequencing data using Cuffdiff and DESeq and the corresponding qRT-PCR data. The heat map visualises the expression of 37 regulated immune genes 12 h after pricking of larvae and workers with a 1:1 mix of Gram-negative and Gram-positive bacteria in case of Illumina sequencing (Cuffdifflog2FC / DeSeq log2FC). The corresponding qRT-PCR analysis was performed separately in larvae and workers revealing a stage-specific gene regulation after immune challenge (log2(x-fold change L2 + W2)). Up and down-regulation are colour coded as given in the key highlighting common directions of gene regulation in the different data sets

Further validation of differential expression of 37 of the genes mentioned above was performed using qRT-PCR analysis. Of these 37 genes 15 were up-regulated at least two-fold, and 12 genes down-regulated in the Illumina data. For this analysis we differentiated between immune challenged larvae and adult animals instead of using pooled developmental stages which had been used for generation of the Illumina data. In general, the qRT-PCR analysis confirmed the Illumina data showing that the selected genes are regulated in the same direction in both larvae and workers, or at least in one of the two developmental stages. The corresponding heat map (Fig. 9) visualises the comparison of immune gene expression results obtained by qRT-PCR or Illumina sequencing. In Table S10 (Additional file 13) the respective x-fold changes as well as p- or *q*-values of these data are shown in detail. The differences between the expression data of immune-related genes gained by Illumina sequencing or qRT-PCR may largely be explained by the fact that the change in expression of several genes after immune-challenge differs substantially between larvae and workers. For example, expression of the genes MPI (*metalloproteinase inhibitor*), SOCS2 (*suppressor of cytokine signaling 2*), cact1 (*cactus*), transf (*transferrin*), PHR (*parathyroid peptide receptor*), ester (*esterase FE4*), PGRP-LB, PGRP-SA, hp67112 (hypothetical protein, Cflo_N_g6748t1), *hymenoptaecin*, and *thioester-containing protein 1* (TEP1) was strongly induced in immune-challenged larvae, but only weakly or not at all in workers (Fig. 9; Additional file 13: Table S10). This is in agreement with previous reports on other insects which indicated that the immune response of larvae might differ from that of adults in holometabolous insects including workers in social insects [79–81]. Thus, in *C. floridanus* immune induction of gene expression appeared to be much stronger in larvae than in workers. At first glance this is somewhat surprising, since ant larvae are constantly cared for and groomed by nurse workers within the protected nest environment, while in particular foraging adult workers should be exposed more frequently and intensely to a feculent environment. However, larvae may be more vulnerable to pathogen infections due to their relatively thin and soft cuticle and the inability to groom themselves. Therefore, a highly responsive immune gene regulation in particular in larvae may contribute to a long term colony success by ensuring a continuous supply of a large number of healthy offspring. In fact, recent infection experiments with *C. pennsylvanicus* larvae indicated that their individual immune response is important and brood care by nurses does not alleviate the individual immune competence of immature stages [82]. On the other hand, brood care is also of prime importance since it was shown recently by cross-fostering experiments that in the ant *Formica selysi* the colony origin of care taker animals contributed to resistance of freshly eclosed animals against an entomopathogenic fungus [83]. In *A. mellifera* it was also shown that developmental stages differ in immunocompetence – larvae and pupae had the highest haemocyte counts while adult workers had the strongest phenoloxidase activity [84].

The gene most strongly induced after immune challenge should also be mentioned here. This gene (Cflo_N_g6748t1) encodes a protein of unknown function. qRT-PCR reveals a more than 700 fold induction after immune challenge in larvae, but only a very moderate (3-fold) induction in workers (Fig. 9; Additional file 13: Table S10). Due to its extreme expression pattern this protein might play an important role in the immune defence of larvae and might merit future attention. Orthologs of this gene are also found in the other ants, in wasps (*N. vitripennis* and *Micropolitis demolitor*) and in the termite *Zootermopsis nevadensis*.

Based on an orthology analysis six common differentially expressed ‘immune genes’ were found when comparing the *C. floridanus* data with a recently published dataset of immune-stimulated honey bees [85]. These genes encode known immune related proteins such as the serine protease Stubble, NF-kappa-B inhibitor Cactus, Nuclear factor NF-kappa-B p110 subunit Relish, leukocyte elastase inhibitor (Serpin), Tyrosine 3-monooxygenase (involved in melanisation and proPO pathway in *Manduca sexta* [86]), and a protein NPC2 homolog (involved in microbial recognition in *Drosophila melanogaster* [87]).

Several genes down-regulated after immune challenge were identified (Fig. 9, Additional file 13: Table S10). Among these genes several encode proteins involved in digestion (e.g. Chymotrypsin, Lipase) and storage (e.g. Hexamerin), which were already described to be down-regulated upon immune-challenge in other insects. This indicates that during infection insects seem to temporarily shut down digestion and synthesis of non-essential proteins in order to use resources for costly defence reactions [88–92].

### Comparison of *C. floridanus* immune proteins with other genome sequenced ants, *A. mellifera*, *N. vitripennis* and *D. melanogaster*

**Fig. 10:**
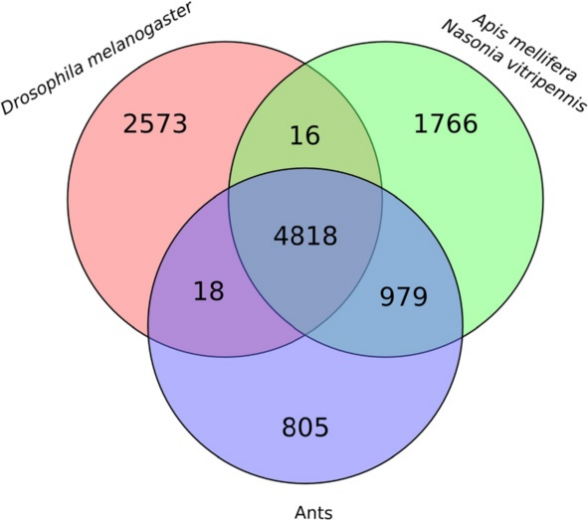
Comparison of insect proteomes. Specific numbers of shared orthologous clusters are indicated (black numbers) comparing the proteomes of 11 insect species (eight ant species (blue) as well as *A. mellifera*, *N. vitripennis* (green) and *D. melanogaster* (red)

Orthologous clusters were identified using OrthoMCL analysis on the proteomes of 11 insect species (Additional file 14: Table S11). OrthoMCL clustering included the eight ant species as well as *A. mellifera*, *N. vitripennis* and *D. melanogaster.* These proteomes clustered into 18,763 groups from 188,092 protein sequences. All eight ant species cluster into 6620 ortholog groups (Fig. 10; details in Additional file 15: Table S12). Looking at the species distribution we found that 4818 groups were shared by all the species analysed, while 5797 groups were shared by all hymenopterans. 79 groups represent genes that are conserved exclusively among the eight ant species (Additional file 16: Table S13). We further parsed the 18,763 groups with perl scripts to reveal the presence of orthologs of *C. floridanus* immune proteins in selected species. Table S14 (Additional file 17) lists the identified immune proteins of *C. floridanus* and their orthologs, if present in the other sequenced ants, *A. mellifera* and *N. vitripennis*. Nine differentially regulated genes encoding putative immune-related proteins of *C. floridanus* do not have any homologs in other insects. KOG annotations of these proteins, listed in Table S15 (Additional file 18), reveal features of some of these proteins including the presence of signal peptides, a chemosensory domain and a DNA-binding domain. Additionally, in a recent study Hamilton and co-workers reported Cathepsin D as a protein that contributes to social immunity in *Camponotus pennsylvanicus* [12]. *C. floridanus* also encodes an ortholog of Cathepsin D (Cflo_N_g9172t1) and it will be interesting to investigate a general role of this protein in social immunity in the future.

## Conclusions

We achieved an improved annotation and detailed analysis of the immune gene repertoire of *C. floridanus* based on the previously published genome sequence of *C. floridanus* and on an Illumina based transcriptome analysis of immune challenged larvae and worker animals. This analysis allowed us to extend the previously annotated protein repertoire not only by about 20 % (including splicing variants), but was instrumental in analysing the immune response of *C. floridanus* and to newly identify nine putative *Camponotus-*specific proteins possibly involved in immune functions or stress response. Furthermore, a comparative overview of immune proteins and pathways is presented which distinguishes between generally conserved parts, ant-specific and *Camponotus*-specific additions. This analysis shows that the immune gene repertoire of *C. floridanus* is comparable to that of the other insects. Especially signalling pathways are highly conserved. However, genes encoding PGN recognition proteins and AMPs appear to be present in a reduced number in comparison to solitary insects such as *D. melanogaster* and *N. vitripennis*. Whether this apparent reduction in the number of certain immune genes is related to an increased use of social or external immune measures is not clear, since no attenuated selective pressure on immune genes could be observed in ant genomes, at least on several selected genes [93]. Moreover, the recent establishment of the complete genomes of two bumblebee species and their comparison with other bee genomes including a solitary bee did not reveal any correlation between the number of immune genes and the degree of sociality evolved [94]. Interestingly, the gene expression analysis suggests significant stage specific differences in the immune responsiveness of *C. floridanus* which may be an important feature possibly contributing to colony success, requiring, however, further investigation in the future. The combination of an apparently quite complex innate immune system and social immunity may explain the resilience of these animals against pathogen infestation despite high population densities in their nests and genetic uniformity.

## Methods

### Sample preparation and RNA extraction

For immune-challenge, late larvae (stage L2) and adult minor workers (stage W2) of colony C90 (for stage definitions see [95]) were pricked with a minutiae needle (Minutiennadel Sphinx V2A 0.1 x 12 mm, bioform), which was previously dipped into a pellet of heat-killed bacteria (1:1 mix of *Escherichia coli* D31 and *Micrococcus luteus*) which are commonly used for infection experiments in insects. Afterwards immune-challenged animals as well as non-treated control animals were kept in artificial nests for 12 h. Then total RNA was extracted from each five immune-challenged and five untreated L2 and W2 using TRIzol® Reagent (Invitrogen, Carlsbad, CA, USA) and purified through RNeasy mini kit columns (Qiagen, Hilden, Germany) with on-column DNase digestion (RNase-Free DNase Set, Qiagen) as described in the manufacturer’s procedures. RNA concentration and quality were determined on an Agilent 2100 Bioanalyzer using the Agilent RNA 6000 Nano Chip kit (Agilent Technologies, Böblingen, Germany) according to the manufacturer´s instructions (Additional file 19: Figure S4). Equal amounts of total RNA from immune-challenged L2 and W2 as well as from untreated L2 and W2 were mixed and the obtained two RNA samples (immune-challenged and naïve) were further processed and sequenced with an Illumina HiSeq2000 as 2× 50 bp reads by Eurofins MWG Operon (Ebersberg, Germany). The total number of resulting reads was 125,873,897 and 118,142,837, respectively.

### Assembly, detection of new transcripts, and differential gene expression analysis

For the discovery of new transcripts and differential gene expression analysis, the protocol described by Trapnell and co-workers was utilised [96]. All analyses were performed on a HP ProLiant DL580 G5 offering four Intel(R) Xeon(R) CPUs E7440 and 40 Gb of RAM. At the first step the read data of both conditions were mapped onto the reference genome (AEAB01000000 [18]) using Tophat (v2.0.4) [97]. Following this, the programme cufflinks (v2.0.2) was used for the assembly of the expressed transcripts [98]. A merged transcriptome annotation was generated using cuffmerge and the differential gene expression analysis was done by cuffdiff (v2.0.2) and, independently, by DESeq [99]. Cuffdiff algorithm estimates the DEGs at transcript-level resolution by comparing the concentration of transcripts in control and treated samples while DESeq is suitable for identifying significantly differentially expressed genes between two samples based on read counts. The R package cummeRbund (v0.1.3, http://compbio.mit.edu/cummeRbund/) was used for the exploration of differentially expressed genes and for the visualisation of differently spliced genes.

### Validation of differential gene expression results by qRT-PCR

For validation of differential gene expression results obtained by transcriptome analysis we performed qRT-PCR. The samples were prepared as described above RNA samples from untreated and immune-challenged animals were prepared in each case from five late larvae (L2) and five adult minor workers (W2) of six different ant colonies (C90, C96, C152, C79, C264, C132) 12 h post infection. RNA was isolated from treated as well as from untreated animals. For each sample cDNA was produced by reverse transcription from 1 μg of total RNA using the RevertAid First Strand cDNA Synthesis Kit (Fermentas). Resulting cDNA was directly diluted to a final concentration of 10 ng/μl.

Expression of candidate genes was analysed by qRT-PCR separately regarding larvae and workers. Oligonucleotide pairs were designed on the chosen genes with Primer3 v. 0.4.0 [100] to yield products of 120–140 bp with T_m_ values around 56 °C (Additional file 20: Table S16). The qRT-PCR experiments were performed on a StepOnePlus™ Real-Time PCR System (Applied Biosystems, Life Technologies GmbH, Darmstadt, Germany). Therefore, samples contained 1× Absolute™ PerfeCTa™ SYBR® Green FastMix™ (Rox) (Quanta Biosciences, Gaithersburg, MD, USA), gene-specific oligonucleotides (250 nM each), 1 μl of the cDNA and water to a final volume of 20 μl. After 5 min of enzyme activation at 95 °C, 45 cycles of 5 s denaturation at 95 °C, 10 s of annealing at 56 °C and 20 s of extension at 60 °C were run. Fragment specificity was checked in melting curves and each biological sample was run in duplicate in the qRT-PCR. Results were averaged and relative transcription levels were calculated by the ddCt method [101] using *ribosomal protein L32* (*rpL32*) as reference gene.

Statistical analysis was performed using Statistica v10 enterprise ×64. A *t*-test was used to test whether the relative gene expression of six biological replicates of infected animals (dCT Immune) differs significantly from relative gene expression of control animals (dCT Control). A *p*-value <0.05 distinguishes a gene as being significantly down- or up-regulated when down-regulation means a ratio <0.5 and up-regulation a ratio >2. Results of the statistical analyses are given in Additional file 21: Table S17.

### Identifying repetitive elements

To delineate various repetitive elements present in the *C. floridanus* genome, RepeatMasker v 4.0 [102] was applied using a custom library comprising a combination of Repbase library [32] and the *de novo* repeat library customised for the *C. floridanus* genome constructed with RepeatModeler [103] with default parameters.

### Gene annotation

Gene models were predicted with the extended version of Generalized Hidden Markov Model (GHMM) based *ab initio* predictor Augustus v2.7 [104–106]. We constructed a high-quality training set consisting of 330 genes for the generation of *C. floridanus* specific parameters for the splice site signals, length distributions, nucleotide composition of exons, introns and intergenic regions. High-confidence gene-models were extracted by combination of a training gene set derived from (a) PASA2 release 2013-06-05 [107] by alignment of cufflinks assembled transcripts on *C. floridanus* genome using GMAP [108] aligner, (b) Cegma v2.4 [109] which uses ortholog identification of a set of accurately annotated 458 eukaryotic core proteins in the test genome followed by determination of exon-intron structure using a combination of GeneWise [110], HMMER [111] and GeneID [112], and (c) Scipio v1.4 [113] by aligning randomly selected *C. floridanus* proteins from different GO categories on the *C. floridanus* genome with BLAT [114] and determining correct exon-intron junctions by hit refinements and filtering with Scipio. We further generated extrinsic evidence (hints) for Augustus predictions from different sources, in particular (a) the raw Illumina reads and (b) the assembled transcripts as well as (c) all EST evidence. Finally, to predict gene structures and estimate alternative transcripts, Augustus predictions with hints were performed on the repeat-masked *C. floridanus* genome using the tuned optimised parameters of *C. floridanus*. Detailed sequence analysis of all genes and proteins included different software, databanks and protocols as described previously [115].

### Reconstruction of immune signalling pathways

The unidirectional and bidirectional interactions among the components of Toll, Jak-Stat, IMD and JNK pathway of *D. melanogaster* were mined individually from three pathway databases KEGG [116], FlyReactome (http://fly.reactome.org/) and INOH [117] followed by extensive manual curations from scientific literatures. The proteins and connectivity information were translated into comprehensive immune signalling networks of *D. melanogaster*. Proteins involved in networks were retrieved from the Entrez protein database available at the National Center for Biotechnology Information (NCBI) (http://ncbi.nlm.nih.gov/) and chosen as queries to perform BlastP search [118] against *C. floridanus* protein sequences available in Entrez and Uniprot databases [119]. Subsequently, we performed domain analysis with PFAM search [120] for the immune related protein sequences of both of the organisms and accessed the relative domain conservation by global pairwise alignments. The identified homologous immune proteins were mapped onto reconstructed immune signalling networks of *D. melanogaster* and pathway annotations were transferred if any of the two immune proteins in *C. floridanus* had corresponding interacting homologs.

### Identification of AMPs

The creation of used HMMs of AMPs has been described elsewhere [121]. Briefly, in the AMPer database [121] 1045 mature peptides associated with known antimicrobial activities were considered to create 146 HMMs of mature peptides. All the 146 HMMs of mature peptides were retrieved from the AMPer resource. We further employed HMMsearch module of HMMER3 [121] with an *E*-value threshold of 1e-03 to scan all the protein isoforms obtained from re-annotation of *C. floridanus* against HMMs of AMPs. In order to remove the homologous peptides in resulting datasets, a cut-off threshold of 90 % was imposed by using the CD-HIT programme [122]. Furthermore, using known APMs of other insects as a query sequence BlastP and tBlastn, searches were conducted to identify additional AMPs.

### Identification of immune effectors

To detect the presence of potential immune effectors including chitinase, lysozymes, prophenoloxidase, nitric oxide synthase, glutathione S-transferase, TEPs and turandots in *C. floridanus* we implemented the BlastP search using the effector sequences from several insects (*D. melanogaster*, *A. mellifera*, *Anopheles gambiae*, *Bombyx mori*, *Manduca sexta*, *Aedes aegypti* and *Phaedon cochleariae*) as a query. The GenBank accession numbers of used query sequences are given in Additional file 22: Table S18.

### Orthology analysis

We employed OrthoMCLv2.0.9 [123] to examine the presence of orthologs of *C. floridanus* immune proteins in the proteomes from seven other sequenced ant genomes, including *A. cephalotes* (leafcutter ant), *A. echinatior* (Panamanian leafcutter ant), *P. barbatus* (red harvester ant), *H. saltator* (Jerdon’s jumping ant), *L. humile* (Argentine ant), *S. invicta* (red fire ant), *C. biroi* (clonal raider ant), *A. mellifera* (honeybee), *N. vitripennis* (a parasitic solitary wasp) and *D. melanogaster* (model insect). OrthoMCL pipeline integrates all-versus-all Blast similarity results and Markov clustering algorithm (MCL) to construct putative orthologous groups, including recent paralogs, across multiple taxa. Blast *e*-value and MCL inflation index was set to 1e-05 and 1.5 respectively for OrthoMCL clustering.

### Availability of supporting data

Additional data for download can be retrieved at www.bioinfo.biozentrum.uni-wuerzburg.de/computing/Camponotus. These files are: *Camponotus floridanus* augustus gene annotations (.gff format), *Camponotus floridanus* transcriptome gene coordinates (.gff format), *Camponotus floridanus* transcripts for the translated regions (fasta format) and *Camponotus floridanus* predicted proteins (fasta format) for the gene set. All files are zipped to save space. Furthermore, the raw data of the transcriptome sequences (just the Illumina reads, no annotation) are deposited in the NCBI bioproject ID263478 Accession: PRJNA263478 to be released upon acceptance of the manuscript. These data have the NCBI accession numbers [GenBank:SRR1609918] for the immune challenged and [GenBank:SRR1609919] for the unchallenged animals.

## Additional files

Additional file 1: Table S1. Summary of genes predicted with Augustus run on the repeat masked *C. floridanus* genome. Additional file 2: Table S2. Accuracy of trained Augustus on *C. floridanus* test set sequences. Additional file 3: Figure S1. Differences in pfam protein domains as deduced from the previous and the new *C. floridanus* genome annotation. Additional file 4: Figure S2. Categorisation of 7143 proteins *of Camponotus floridanus* v3.3set in GO terms (level 2) for (A) biological process, (B) molecular function, and (C) cellular component using a filter score *e*-value cutoff of 1e-5. Additional file 5: Table S3. Global changes in level 2 GO class assignment of new and previously annotated Cflov3.3 proteins for the following GO categories: biological process, molecular function and cellular component. Additional file 6: Table S4. Functional annotation of highly conserved immune proteins shared by *D. melanogaster*, *A. mellifera*, *N. vitripennis* and *C. floridanus*. Additional file 7: Table S5. Structural and functional annotation of *C. floridanus* immune-related proteins. Additional file 8: Table S6. List of serine proteases and serine protease inhibitors of *C. floridanus*. Additional file 9: Table S7. List of putative chitinases of *C. floridanus* and distribution of conserved motifs in their deduced amino acid sequences*.*
Additional file 10: Table S8. Number of chitinases, glutathione S-transferases, nitric oxide synthases, thioester-containing proteins, lysozymes and prophenoloxidases encoded by *C. floridanus*, other ants, *A. mellifera*, *N. vitripennis* and *D. melanogaster*. Additional file 11: Figure S3. Phylogenetic relationship between GSTs of *C. floridanus* and of other insects as inferred using the Neighbor Joining algorithm [124]. The statistical reliability of the phylogenetic tree was tested by bootstrap analyses with 10,000 replications. The topology is based on a 50 % condensed tree obtained by bootstrap analysis. The percentage of replicate trees in which the associated nodes clustered together in the bootstrap test is shown next to the branches. Species abbreviations occur after the GenBank accession numbers are as follows: Dm = *Drosophila melanogaster*, Ag = *Anopheles gambiae*, Am = *Apis mellifera*, Bm = *Bombyx mori*, Aa = *Aedes aegypti*, Cf = *Camponotus floridanus*. Evolutionary analyses were conducted in MEGA v5.1 [125]. Additional file 12: Table S9. Complete list of genes of *C. floridanus* which are differentially expressed after immune challenge. The genes listed were retrieved based on the analysis of the Illumina sequencing data using the programs Cufflinks and DESeq. The table shows significantly up-regulated and down-regulated genes 12 h after picking of larvae and workers with a 1:1 mix of Gram-negative and Gram-positive bacteria. Cufflinks analysis resulted in new gene identifiers (XLOCs), which correspond to one or more of the originally annotated genes (EAGs). Additional file 13: Table S10. Genes differentially expressed (DEGs) after immune challenge. The table presents a comparison of expression data of 37 selected immune genes based on the analysis of the Illumina sequencing data (Cuffdiff and DESeq) and the corresponding qRT-PCR data. Significance of resulting log2(x-fold change) values is given by asterisks (* *p*-value/*q*-value ≤0.05; ** ≤0.01; *** ≤0.001; n.s. for non-significant results) Additional file 14: Table S11. The source of proteomes of the eleven insect species included in our OrthoMCL analysis. Additional file 15: Table S12. Comparison of orthologous groups identified by OrthoMCL using an inflation index of 1.5. Additional file 16: Table S13. Conserved orthologous groups shared among all eight sequenced ant species identified by OrthoMCL analysis. Additional file 17: Table S14. List of the immune-related proteins of *C. floridanus* and their orthologs, if also present in the other sequenced ants, and in *A. mellifera* and *N. vitripennis*. Additional file 18: Table S15. List of *C. floridanus* specific hypothetical genes expressed differentially after immune challenge including KOG annotations. Additional file 19: Figure S4. Quality control of total RNA samples. (A) The image shows a total RNA gel like-image produced by the Agilent 2100 Bioanalyzer. Lane 1: RNA from *C. floridanus* workers and larvae (1:1 mix) at 12 h after bacterial challenge, Lane 2: RNA from untreated workers (W2) and larvae (L2) (1:1 mix). (B) Electrophoretic profiles of RNA from immune-challenged (1) and untreated (2) W2 and L2 (1:1 mix). [FU]: Fluorescence units, [s]: seconds. Additional file 20: Table S16. List of oligonucleotides used for validation of changes of gene expression after immune challenge by qRT-PCR. Additional file 21: Table S17. Statistical analysis of qRT-PCR results: The tables A (larvae) and B (adult workers) show normalised values (dCT) of immunised and control animals of six different colonies when *rpL32* was used as the housekeeping gene. These values were used in a *t*-test to determine significant differential gene expression (mean ratio as x-fold expression also given here). A *p*-value <0.05 distinguishes a gene as being significantly down- or up-regulated (highlighted in red). Additional file 22: Table S18. Accession numbers of the sequentially used query sequences from different insects.
