# Supplementary material for: Scrutinizing the immune defence inventory of Camponotus floridanus applying total transcriptome sequencing
Source: BMC Genomics. 2015 Jul 22;16(1):540. doi: 10.1186/s12864-015-1748-1 (PMC4508827; doi:10.1186/s12864-015-1748-1)
Supplement: Additional file 1: Table S1. — Summary of genes predicted with Augustus run on the repeat masked C. floridanus genome. [file 12864_2015_1748_MOESM1_ESM.docx]

**Additional File 1: Table S1:** Accuracy of trained Augustus on *C. floridanus* test set sequences.

|  | **Base level** | | | **Exon level** | | |
| --- | --- | --- | --- | --- | --- | --- |
| **Program** | Sensitivity (Sn) | Specificity (Sp) | (Sn+Sp)/2 | Sensitivity (Sn) | Specificity (Sp) | (Sn+Sp)/2 |
| Augustus with hints | 0.953 | 0.906 | 0.9295 | 0.82 | 0.796 | 0.808 |
