# Supplementary material for: Scrutinizing the immune defence inventory of Camponotus floridanus applying total transcriptome sequencing
Source: BMC Genomics. 2015 Jul 22;16(1):540. doi: 10.1186/s12864-015-1748-1 (PMC4508827; doi:10.1186/s12864-015-1748-1)
Supplement: Additional file 2: Table S2. — Accuracy of trained Augustus on C. floridanus test set sequences. [file 12864_2015_1748_MOESM2_ESM.docx]

**Additional File 2: Table S2:** Summary of genes predicted with Augustus run on repeat masked *C. floridanus* genome.

| **Number of genes** | **Count of alternative transcripts** |
| --- | --- |
| 13703 | 1 |
| 1410 | 2 |
| 346 | 3 |
| 106 | 4 |
| 36 | 5 |
| 15 | 6 |
| 10 | 7 |
| 3 | 8 |
| None | 9 |
| 2 | 10 |
| None | >10 |
| # Total - 15631 genes | # Total - 18369 transcripts |
