# Supplementary figures and images for: Scrutinizing the immune defence inventory of Camponotus floridanus applying total transcriptome sequencing
Source: BMC Genomics. 2015 Jul 22;16(1):540. doi: 10.1186/s12864-015-1748-1 (PMC4508827; doi:10.1186/s12864-015-1748-1)

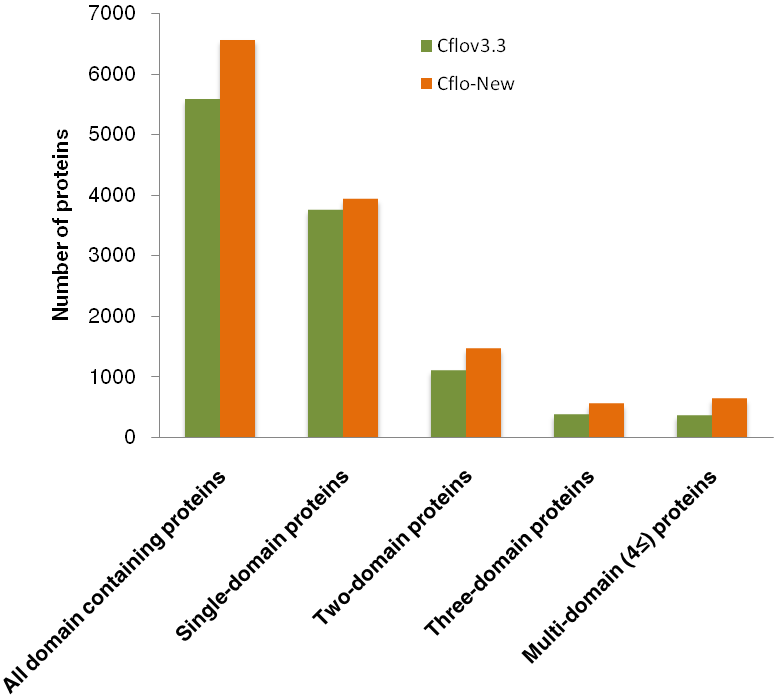

Supplement: Additional file 3: Figure S1. — Differences in pfam protein domains as deduced from the previous and the new C. floridanus genome annotation. [file 12864_2015_1748_MOESM3_ESM.png]

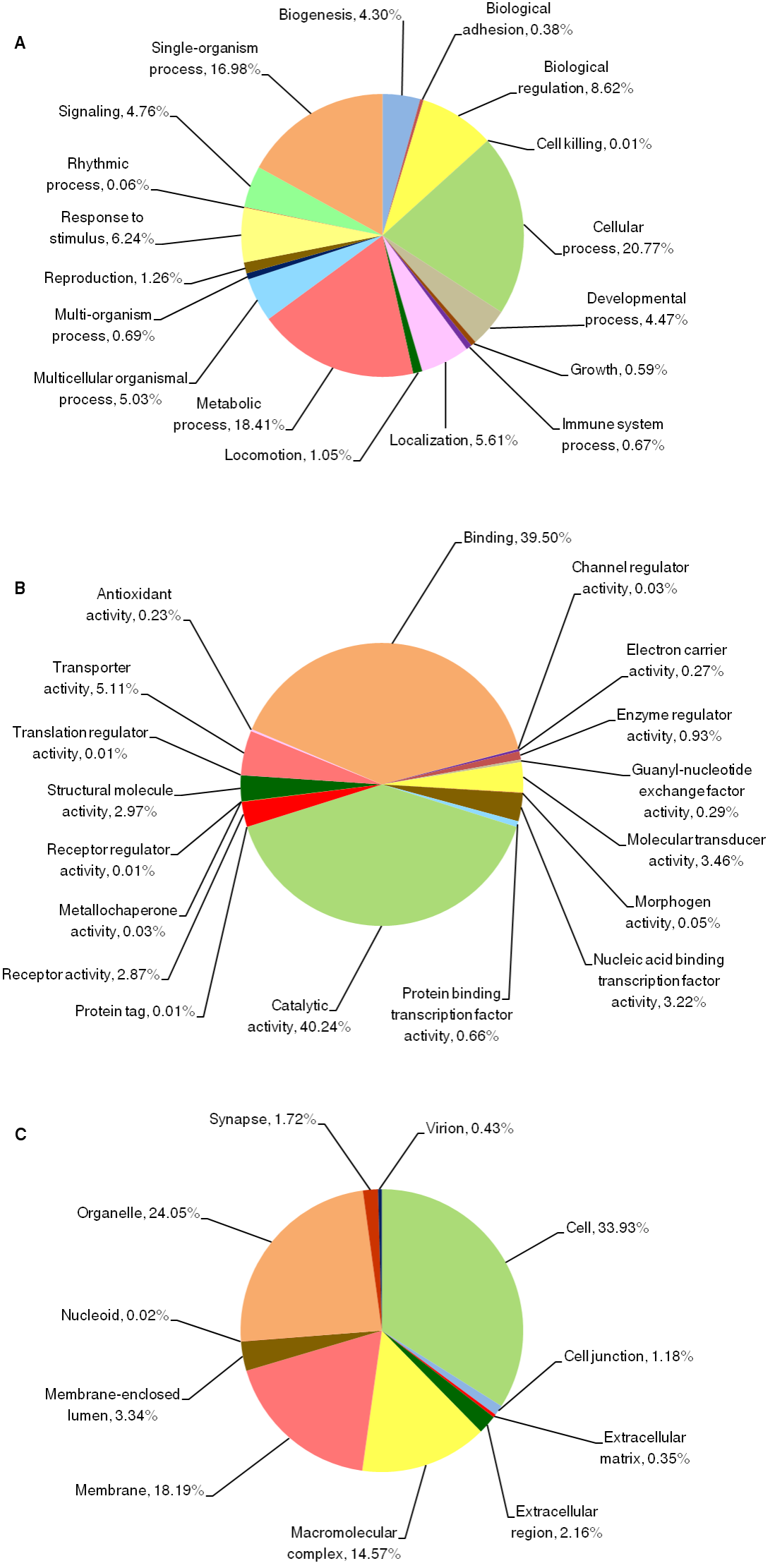

Supplement: Additional file 4: Figure S2. — Categorisation of 7143 proteins of Camponotus floridanus v3.3set in GO terms (level 2) for (A) biological process, (B) molecular function, and (C) cellular component using a filter score e-value cutoff of 1e-5. [file 12864_2015_1748_MOESM4_ESM.png]

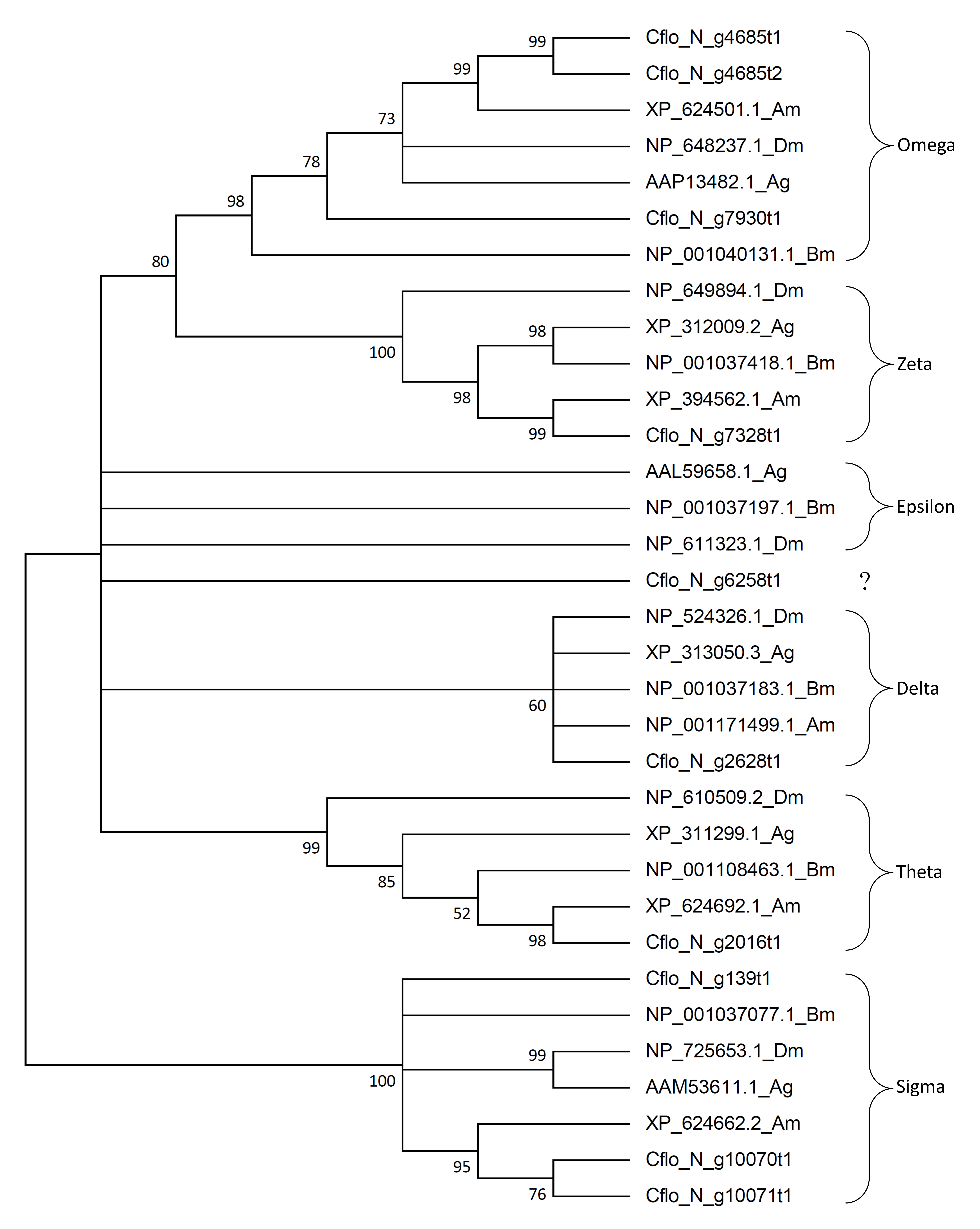

Supplement: Additional file 11: Figure S3. — Phylogenetic relationship between GSTs of C. floridanus and of other insects as inferred using the Neighbor Joining algorithm [124]. The statistical reliability of the phylogenetic tree was tested by bootstrap analyses with 10,000 replications. The topology is based on a 50 % condensed tree obtained by bootstrap analysis. The percentage of replicate trees in which the associated nodes clustered together in the bootstrap test is shown next to the branches. Species abbreviations occur after the GenBank accession numbers are as follows: Dm = Drosophila melanogaster, Ag = Anopheles gambiae, Am = Apis mellifera, Bm = Bombyx mori, Aa = Aedes aegypti, Cf = Camponotus floridanus. Evolutionary analyses were conducted in MEGA v5.1 [125]. [file 12864_2015_1748_MOESM11_ESM.png]

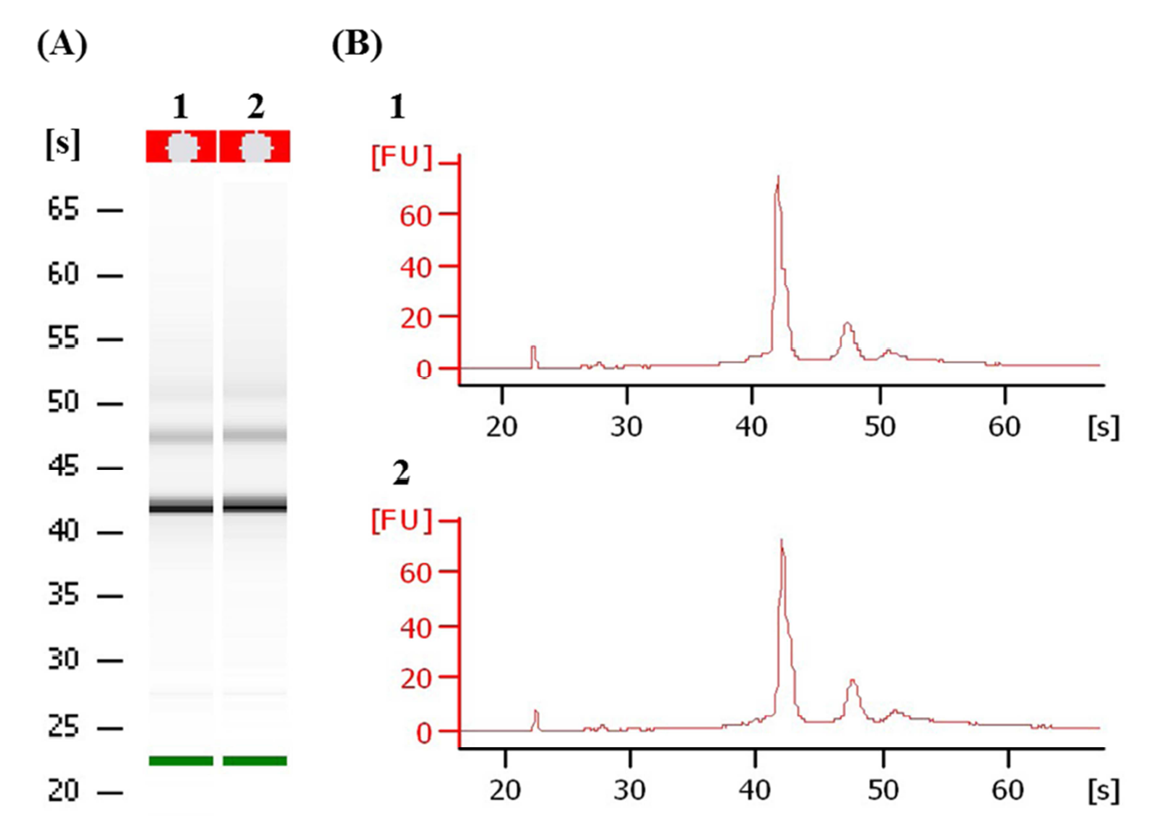

Supplement: Additional file 19: Figure S4. — Quality control of total RNA samples. (A) The image shows a total RNA gel like-image produced by the Agilent 2100 Bioanalyzer. Lane 1: RNA from C. floridanus workers and larvae (1:1 mix) at 12 h after bacterial challenge, Lane 2: RNA from untreated workers (W2) and larvae (L2) (1:1 mix). (B) Electrophoretic profiles of RNA from immune-challenged (1) and untreated (2) W2 and L2 (1:1 mix). [FU]: Fluorescence units, [s]: seconds. [file 12864_2015_1748_MOESM19_ESM.png]
