# Supplementary material for: Scrutinizing the immune defence inventory of Camponotus floridanus applying total transcriptome sequencing
Source: BMC Genomics. 2015 Jul 22;16(1):540. doi: 10.1186/s12864-015-1748-1 (PMC4508827; doi:10.1186/s12864-015-1748-1)
Supplement: Additional file 5: Table S3. — Global changes in level 2 GO class assignment of new and previously annotated Cflov3.3 proteins for the following GO categories: biological process, molecular function and cellular component. [file 12864_2015_1748_MOESM5_ESM.docx]

**Additional File 5: Table S3:** Global changes in level 2 GO class assignment of new and Cflov3.3 proteins for GO categories biological process, molecular function and cellular component.

| **Biological process (Level 2 GO annotation)** | | |
| --- | --- | --- |
|  | Cflo-New  (13354 terms) | Cflov3.3  (10819 terms) |
| Cell killing | 2 | 2 |
| Multicellular organismal process | 748 | 545 |
| Rhythmic process | 12 | 7 |
| Biological adhesion | 68 | 42 |
| Multi-organism process | 84 | 75 |
| Localization | 775 | 608 |
| Metabolic process | 2267 | 1992 |
| Signaling | 695 | 515 |
| Reproduction | 185 | 137 |
| Cellular process | 2709 | 2248 |
| Response to stimulus | 866 | 676 |
| Biological regulation | 1215 | 933 |
| Immune system process | 73 | 73 |
| Growth | 108 | 64 |
| Single-organism process | 2251 | 1838 |
| Developmental process | 686 | 484 |
| Locomotion | 183 | 114 |
| Biogenesis | 627 | 466 |
|  |  |  |
| **Molecular function (Level 2 GO annotation)** | | |
|  | Cflo-New  (6088 terms) | Cflov3.3  (5141 terms) |
| Receptor regulator activity | 1 | 1 |
| Structural molecule activity | 150 | 153 |
| Translation regulator activity | 1 | 1 |
| Transporter activity | 351 | 263 |
| Antioxidant activity | 13 | 12 |
| Binding | 2380 | 2031 |
| Channel regulator activity | 3 | 2 |
| Electron carrier activity | 13 | 14 |
| Enzyme regulator activity | 66 | 48 |
| Guanyl-nucleotide exchange factor activity | 24 | 15 |
| Molecular transducer activity | 225 | 178 |
| Morphogen activity | 1 | 3 |
| Nucleic acid binding transcription factor activity | 199 | 166 |
| Protein binding transcription factor activity | 41 | 34 |
| Catalytic activity | 2440 | 2069 |
| Protein tag | 2 | 1 |
| Receptor activity | 178 | 148 |
| Metallochaperone activity | 0 | 2 |
|  |  |  |
| **Cellular component (Level 2 GO annotation)** | | |
|  | Cflo-New  (5441 terms) | Cflov3.3  (4568 terms) |
| Cell | 1836 | 1550 |
| Cell junction | 98 | 54 |
| Extracellular matrix | 22 | 16 |
| Extracellular region | 93 | 99 |
| Macromolecular complex | 798 | 666 |
| Membrane | 1024 | 831 |
| Membrane-enclosed lumen | 173 | 153 |
| Nucleoid | 1 | 1 |
| Organelle | 1250 | 1099 |
| Synapse | 124 | 79 |
| Virion | 22 | 20 |
