# Supplementary material for: Scrutinizing the immune defence inventory of Camponotus floridanus applying total transcriptome sequencing
Source: BMC Genomics. 2015 Jul 22;16(1):540. doi: 10.1186/s12864-015-1748-1 (PMC4508827; doi:10.1186/s12864-015-1748-1)
Supplement: Additional file 9: Table S7. — List of putative chitinases of C. floridanus and distribution of conserved motifs in their deduced amino acid sequences. [file 12864_2015_1748_MOESM9_ESM.docx]

**Additional File 9: Table S7:** List of putative chitinases of *C. floridanus* and distribution of conserved motifs in their deduced amino acid sequences*.*

| Accession no. | Position of conserved motifs | | | |
| --- | --- | --- | --- | --- |
|  | Motif I  KXXXXXGGW | Motif II  FDGXDLDWEYP | Motif III  MXYDXXG | Motif IV  GXXXWXXDXD |
| Cflo_N_g2277t1 | 82-90 | NF | 194-200 | NF |
| Cflo_N_g8995t1 | 245-253 | NF | 357-363 | 491-500 |
| Cflo_N_g14547t1 | NF | NF | NF | NF |
| Cflo_N_g10513t1 | 1190-1198, 1601-1609, 2122-2130 | 1232-1242 | 508-514, 1304-1310, 1719-1725 | 188-197, 648-657, 1450-1459, 1865-1874, 2391-2400 |
| Cflo_N_g10891t1 | 156-164, 569-577 | NF | 259-265, 684-690 | 405-414, 831-840 |
| Cflo_N_g260t1 | NF | NF | NF | NF |
| Cflo_N_g12931t1 | 101-109 | 142-152 | 217-223 | 370-379 |
| Cflo_N_g8158t1 | 59-67 | 100-110 | NF | 330-339 |
| Cflo_N_g8158t2 | 59-67 | 100-110 | NF | 330-339 |
| Cflo_N_g7573t1 | NF | NF | NF | NF |
| Cflo_N_g9837t1 | NF | NF | NF | 220-229 |
| Cflo_N_g9838t1 | NF | NF | NF | NF |
| Cflo_N_g10512t1 | NF | NF | NF | NF |

Note – Gene Cflo_N_g8158 codes two alternative spliced protein products Cflo_N_g8158t1 and Cflo_N_g8158t2. Abbreviation NF – not found.
