# Supplementary material for: Scrutinizing the immune defence inventory of Camponotus floridanus applying total transcriptome sequencing
Source: BMC Genomics. 2015 Jul 22;16(1):540. doi: 10.1186/s12864-015-1748-1 (PMC4508827; doi:10.1186/s12864-015-1748-1)
Supplement: Additional file 10: Table S8. — Number of chitinases, glutathione S-transferases, nitric oxide synthases, thioester-containing proteins, lysozymes and prophenoloxidases encoded by C. floridanus, other ants, A. mellifera, N. vitripennis and D. melanogaster. [file 12864_2015_1748_MOESM10_ESM.docx]

|  | **Chitinases** | **Glutathione-**  **S-transferases**  **(GSTs)** | **Nitric oxide synthases**  **(NOS)** | **Thioester- containing proteins (TEPs)** | **Lysozymes** | **Phenoloxidases** |
| --- | --- | --- | --- | --- | --- | --- |
| ***Camponotus floridanus*** | **13** | **10** | **1** | **4** | **3** | **1** |
| ***Atta***  ***cephalotes*** | **10** | **11** | **2** | **4** | **3** | **1** |
| ***Acromyrmex echinatior*** | **11** | **11** | **1** | **3** | **3** | **1** |
| ***Pogonomyrmex barbatus*** | **10** | **10** | **1** | **3** | **2** | **1** |
| ***Harpegnathos saltator*** | **12** | **9** | **1** | **3** | **2** | **1** |
| ***Linepithema humile*** | **9** | **8** | **1** | **3** | **2** | **1** |
| ***Solenopsis***  ***invicta*** | **9** | **9** | **1** | **4** | **3** | **1** |
| ***Cerapachys***  ***biroi*** | **9** | **10** | **1** | **3** | **2** | **2** |
| ***Apis***  ***mellifera*** | **9** | **10** | **1** | **3** | **3** | **1** |
| ***Nasonia vitripennis*** | **13** | **11** | **2** | **3** | **2** | **3** |
| ***Drosophila melanogaster*** | **8** | **20** | **1** | **5** | **7** | **3** |

**Additional File 10: Table S8**
