# Supplementary material for: Scrutinizing the immune defence inventory of Camponotus floridanus applying total transcriptome sequencing
Source: BMC Genomics. 2015 Jul 22;16(1):540. doi: 10.1186/s12864-015-1748-1 (PMC4508827; doi:10.1186/s12864-015-1748-1)
Supplement: Additional file 14: Table S11. — The source of proteomes of the eleven insect species included in our OrthoMCL analysis. [file 12864_2015_1748_MOESM14_ESM.docx]

**Additional File 14: Table S11:** The source of proteomes of 11 insect species for OrthoMCL analysis.

| Species | URL |
| --- | --- |
| *Camponotus floridanus* | This manuscript. |
| *Atta cephalotes* | http://hymenopteragenome.org/drupal/sites/hymenopteragenome.org.atta/files/data/acep_OGSv1.2_pep.fa.gz |
| *Acromyrmex echinatior* | http://hymenopteragenome.org/drupal/sites/hymenopteragenome.org.acromyrmex/files/data/aech_OGSv3.8_pep.fa.gz |
| *Pogonomyrmex barbatus* | http://hymenopteragenome.org/drupal/sites/hymenopteragenome.org.pogo/files/data/pbar_OGSv1.2_pep.fa.gz |
| *Harpegnathos saltator* | http://hymenopteragenome.org/drupal/sites/hymenopteragenome.org.harpegnathos/files/data/hsal_OGSv3.3_pep.fa.gz |
| *Linepithema humile* | http://hymenopteragenome.org/drupal/sites/hymenopteragenome.org.linepithema/files/data/lhum_OGSv1.2_pep.fa.gz |
| *Solenopsis invicta* | http://hymenopteragenome.org/drupal/sites/hymenopteragenome.org.solenopsis/files/data/sinv_OGSv2.2.3_pep.fa.gz |
| *Cerapachys biroi* | http://www.ncbi.nlm.nih.gov/protein?term=txid443821[Organism] |
| *Apis mellifera* | ftp://ftp.ensemblgenomes.org/pub/release-22/metazoa/fasta/apis_mellifera/pep/Apis_mellifera.Amel4.0.22.pep.all.fa.gz |
| *Nasonia vitripennis* | ftp://cegg.unige.ch/OrthoDB6/FASTA/METAZOA/NVITR.Nasonia_vitripennis.fas.gz |
| *Drosophila melanogaster* | ftp://cegg.unige.ch/OrthoDB6/FASTA/METAZOA/DMELA.Drosophila_melanogaster.fas.gz |
