# Supplementary material for: Scrutinizing the immune defence inventory of Camponotus floridanus applying total transcriptome sequencing
Source: BMC Genomics. 2015 Jul 22;16(1):540. doi: 10.1186/s12864-015-1748-1 (PMC4508827; doi:10.1186/s12864-015-1748-1)
Supplement: Additional file 15: Table S12. — Comparison of orthologous groups identified by OrthoMCL using an inflation index of 1.5. [file 12864_2015_1748_MOESM15_ESM.docx]

**Additional File 15: Table S12:** Comparison of orthologous groups identified by OrthoMCL using inflation index 1.5.

|  | Total sequences | OrthoMCL grouped sequences | OrthoMCL grouped sequences (no inparlogs)* |
| --- | --- | --- | --- |
| # All protein sequences | 188092 | 145047 (77.11%) | 133603 (71.03%) |
| *Camponotus floridanus* | 18369 | 14783 (80.47%) | 13554 (73.78%) |
| *Atta cephalotes* | 18093 | 12812 (70.81%) | 12703 (70.20%) |
| *Acromyrmex echinatior* | 17278 | 13300 (76.97%) | 12372 (71.60%) |
| *Pogonomyrmex barbatus* | 17186 | 13301 (77.39%) | 13077 (76.09%) |
| *Harpegnathos saltator* | 18564 | 14920 (80.37%) | 12280 (66.14%) |
| *Linepithema humile* | 16114 | 13303 (82.55%) | 13040 (89.92%) |
| *Solenopsis invicta* | 16522 | 13427 (81.26%) | 12870 (77.89%) |
| *Cerapachys biroi* | 16679 | 13569 (81.35%) | 12718 (76.25%) |
| *Apis mellifera* | 10991 | 10122 (92.09%) | 9958 (90.60%) |
| *Nasonia vitripennis* | 24369 | 18781 (77.06%) | 12957 (53.17%) |
| *Drosophila melanogaster* | 13927 | 10112 (72.60%) | 8074 (57.97%) |
| # Groups |  | #18763 | #15471 |

*Number of sequences in the groups was determined after removal of the same species in paralog clusters.
