# Supplementary material for: Scrutinizing the immune defence inventory of Camponotus floridanus applying total transcriptome sequencing
Source: BMC Genomics. 2015 Jul 22;16(1):540. doi: 10.1186/s12864-015-1748-1 (PMC4508827; doi:10.1186/s12864-015-1748-1)
Supplement: Additional file 16: Table S13. — Conserved orthologous groups shared among all eight sequenced ant species identified by OrthoMCL analysis. [file 12864_2015_1748_MOESM16_ESM.docx]

**Additional File 16: Table S13:** Conserved orthologous groups shared among all eight sequenced ant species identified by OrthoMCL analysis.

| **ant_ortho_group1:** | aech\|AECH12734-PA aech\|AECH17925-PA hsal\|HSAL12934-PA pbar\|PB21817-PA cbir\|EZA62605.1 lhum\|LH20614-PA cflo\|Cflo_N_g8341t1 sinv\|SINV25411-PA acep\|ACEP12750-PA hsal\|HSAL12935-PA cbir\|EZA62795.1 cflo\|Cflo_N_g8343t1 pbar\|PB26160-PA acep\|ACEP16480-PA lhum\|LH20607-PA acep\|ACEP16484-PA lhum\|LH18177-PA |
| --- | --- |
| **ant_ortho_group2:** | cflo\|Cflo_N_g12492t1 cflo\|Cflo_N_g15599t1 cflo\|Cflo_N_g5490t1 cflo\|Cflo_N_g12875t1 cbir\|EZA48265.1 cbir\|EZA54570.1 sinv\|SINV20108-PA sinv\|SINV25907-PA cflo\|Cflo_N_g1723t1 sinv\|SINV16904-PA acep\|ACEP15729-PA pbar\|PB24705-PA hsal\|HSAL23647-PA pbar\|PB18196-PA pbar\|PB10903-PA lhum\|LH14489-PA aech\|AECH26197-PA |
| **ant_ortho_group3:** | cbir\|EZA47890.1 cbir\|EZA47891.1 cbir\|EZA48269.1 hsal\|HSAL18849-PA hsal\|HSAL18850-PA hsal\|HSAL18851-PA pbar\|PB23955-PA pbar\|PB23956-PA aech\|AECH24025-PA sinv\|SINV12289-PA cflo\|Cflo_N_g9909t1 lhum\|LH26052-PA sinv\|SINV12276-PA acep\|ACEP16219-PA cflo\|Cflo_N_g14970t1 acep\|ACEP11443-PA |
| **ant_ortho_group4:** | cflo\|Cflo_N_g4261t1 cflo\|Cflo_N_g4261t2 hsal\|HSAL18557-PA sinv\|SINV11569-PA pbar\|PB16822-PA lhum\|LH24394-PA pbar\|PB16819-PA cbir\|EZA61171.1 lhum\|LH24393-PA sinv\|SINV11571-PA acep\|ACEP23605-PA aech\|AECH11909-PA acep\|ACEP23609-PA aech\|AECH11907-PA |
| **ant_ortho_group5:** | cbir\|EZA48737.1 cbir\|EZA54256.1 cbir\|EZA54517.1 cbir\|EZA59431.1 sinv\|SINV13111-PA sinv\|SINV13114-PA sinv\|SINV13119-PA aech\|AECH21666-PA cflo\|Cflo_N_g2190t1 pbar\|PB25316-PA lhum\|LH18114-PA hsal\|HSAL16199-PA acep\|ACEP17434-PA |
| **ant_ortho_group6:** | aech\|AECH18554-PA hsal\|HSAL10318-PA cbir\|EZA55323.1 cflo\|Cflo_N_g2904t1 pbar\|PB11951-PA sinv\|SINV24747-PA lhum\|LH13761-PA cflo\|Cflo_N_g2904t2 pbar\|PB11949-PA lhum\|LH13764-PA sinv\|SINV24780-PA acep\|ACEP25679-PA acep\|ACEP25682-PA |
| **ant_ortho_group7:** | cflo\|Cflo_N_g6635t1 cflo\|Cflo_N_g6635t2 cflo\|Cflo_N_g6635t3 cflo\|Cflo_N_g7931t1 cbir\|EZA57636.1 hsal\|HSAL13348-PA pbar\|PB17858-PA lhum\|LH25030-PA acep\|ACEP18911-PA aech\|AECH11637-PA sinv\|SINV17734-PA |
| **ant_ortho_group8:** | cbir\|EZA62371.1 cbir\|EZA62372.1 cflo\|Cflo_N_g1207t3 sinv\|SINV23408-PA hsal\|HSAL20022-PA lhum\|LH18672-PA acep\|ACEP16495-PA sinv\|SINV23119-PA lhum\|LH18673-PA aech\|AECH22870-PA pbar\|PB20272-PA |
| **ant_ortho_group9:** | aech\|AECH21079-PA pbar\|PB20174-PA hsal\|HSAL17061-PA sinv\|SINV20859-PA lhum\|LH16922-PA cbir\|EZA49141.1 cflo\|Cflo_N_g3861t1 pbar\|PB20173-PA sinv\|SINV20884-PA acep\|ACEP22303-PA acep\|ACEP22318-PA |
| **ant_ortho_group10:** | cflo\|Cflo_N_g11725t1 cflo\|Cflo_N_g11725t2 cflo\|Cflo_N_g11725t3 lhum\|LH25682-PA cbir\|EZA56600.1 sinv\|SINV14929-PA pbar\|PB26476-PA hsal\|HSAL16308-PA acep\|ACEP11480-PA aech\|AECH22973-PA |
| **ant_ortho_group11:** | cflo\|Cflo_N_g12014t1 cflo\|Cflo_N_g12014t2 cflo\|Cflo_N_g12014t3 acep\|ACEP12132-PA aech\|AECH27230-PA sinv\|SINV10700-PA lhum\|LH24118-PA cbir\|EZA48930.1 pbar\|PB22643-PA hsal\|HSAL21859-PA |
| **ant_ortho_group12:** | cflo\|Cflo_N_g7451t1 cflo\|Cflo_N_g7451t2 cflo\|Cflo_N_g7451t3 aech\|AECH13032-PA pbar\|PB22553-PA cbir\|EZA52674.1 sinv\|SINV19094-PA hsal\|HSAL16204-PA lhum\|LH23142-PA acep\|ACEP23017-PA |
| **ant_ortho_group13:** | cbir\|EZA47266.1 cbir\|EZA57489.1 cbir\|EZA60699.1 aech\|AECH21701-PA sinv\|SINV16136-PA pbar\|PB13001-PA cflo\|Cflo_N_g4641t1 lhum\|LH21283-PA hsal\|HSAL10273-PA acep\|ACEP12973-PA |
| **ant_ortho_group14:** | pbar\|PB18711-PA pbar\|PB21225-PA sinv\|SINV21922-PA sinv\|SINV21947-PA aech\|AECH19543-PA lhum\|LH12388-PA cbir\|EZA59136.1 cflo\|Cflo_N_g9721t1 hsal\|HSAL14455-PA acep\|ACEP26900-PA |
| **ant_ortho_group15:** | aech\|AECH15662-PA pbar\|PB21003-PA sinv\|SINV11787-PA cflo\|Cflo_N_g7025t1 hsal\|HSAL14751-PA lhum\|LH20079-PA cbir\|EZA57058.1 acep\|ACEP14910-PA cbir\|EZA57057.1 lhum\|LH20079-PB |
| **ant_ortho_group16:** | cflo\|Cflo_N_g10889t1 cflo\|Cflo_N_g10889t2 acep\|ACEP13741-PA aech\|AECH20816-PA sinv\|SINV20335-PA pbar\|PB19043-PA cbir\|EZA55159.1 hsal\|HSAL12822-PA lhum\|LH11371-PA |
| **ant_ortho_group17:** | cflo\|Cflo_N_g12624t2 cflo\|Cflo_N_g12624t3 aech\|AECH20453-PA pbar\|PB23112-PA hsal\|HSAL20352-PA cbir\|EZA61694.1 sinv\|SINV14782-PA lhum\|LH15274-PA acep\|ACEP17640-PA |
| **ant_ortho_group18:** | cflo\|Cflo_N_g14194t1 cflo\|Cflo_N_g14194t2 lhum\|LH13173-PA sinv\|SINV26039-PA cbir\|EZA57801.1 pbar\|PB23945-PA hsal\|HSAL13857-PA acep\|ACEP23850-PA aech\|AECH11513-PA |
| **ant_ortho_group19:** | cflo\|Cflo_N_g3003t1 cflo\|Cflo_N_g3003t2 aech\|AECH19724-PA pbar\|PB18105-PA sinv\|SINV13349-PA lhum\|LH15358-PA hsal\|HSAL18979-PA cbir\|EZA61478.1 acep\|ACEP26149-PA |
| **ant_ortho_group20:** | cflo\|Cflo_N_g3581t1 cflo\|Cflo_N_g3581t2 aech\|AECH19856-PA sinv\|SINV13344-PA lhum\|LH15295-PA pbar\|PB12747-PA cbir\|EZA61539.1 hsal\|HSAL18768-PA acep\|ACEP16420-PA |
| **ant_ortho_group21:** | cflo\|Cflo_N_g3851t1 cflo\|Cflo_N_g3851t2 sinv\|SINV13959-PA pbar\|PB26027-PA cbir\|EZA50385.1 lhum\|LH23701-PA hsal\|HSAL19774-PA acep\|ACEP20090-PA aech\|AECH10959-PA |
| **ant_ortho_group22:** | cflo\|Cflo_N_g4619t1 cflo\|Cflo_N_g4619t2 aech\|AECH11267-PA sinv\|SINV10890-PA pbar\|PB17570-PA cbir\|EZA55753.1 hsal\|HSAL20722-PA lhum\|LH16320-PA acep\|ACEP21334-PA |
| **ant_ortho_group23:** | cflo\|Cflo_N_g6757t1 cflo\|Cflo_N_g6757t2 aech\|AECH10655-PA sinv\|SINV25900-PA pbar\|PB22973-PA cbir\|EZA55918.1 lhum\|LH12798-PA hsal\|HSAL19463-PA acep\|ACEP10236-PA |
| **ant_ortho_group24:** | aech\|AECH10629-PA sinv\|SINV13927-PA pbar\|PB12265-PA cflo\|Cflo_N_g4944t1 cbir\|EZA58700.1 hsal\|HSAL22030-PA acep\|ACEP10267-PA acep\|ACEP10269-PA lhum\|LH11056-PA |
| **ant_ortho_group25:** | aech\|AECH22059-PA cbir\|EZA54813.1 hsal\|HSAL23137-PA lhum\|LH13261-PA aech\|AECH22060-PA sinv\|SINV21478-PA pbar\|PB10783-PA acep\|ACEP15175-PA cflo\|Cflo_N_g10280t1 |
| **ant_ortho_group26:** | aech\|AECH21886-PA cbir\|EZA52221.1 pbar\|PB15981-PA cflo\|Cflo_N_g2150t1 hsal\|HSAL17819-PA sinv\|SINV16403-PA lhum\|LH20636-PA pbar\|PB15982-PA acep\|ACEP26696-PA |
| **ant_ortho_group27:** | aech\|AECH21912-PA sinv\|SINV22844-PA lhum\|LH15844-PA pbar\|PB16033-PA cflo\|Cflo_N_g6108t1 cbir\|EZA49621.1 hsal\|HSAL12764-PA acep\|ACEP24025-PB acep\|ACEP24025-PA |
| **ant_ortho_group28:** | acep\|ACEP12348-PA aech\|AECH13156-PA pbar\|PB15889-PA sinv\|SINV11526-PA cflo\|Cflo_N_g6893t1 cbir\|EZA52840.1 lhum\|LH21522-PA hsal\|HSAL17412-PA |
| **ant_ortho_group29:** | aech\|AECH13324-PA lhum\|LH19844-PA sinv\|SINV16364-PA cflo\|Cflo_N_g6390t1 pbar\|PB19834-PA cbir\|EZA55383.1 hsal\|HSAL12221-PA acep\|ACEP26519-PA |
| **ant_ortho_group30:** | aech\|AECH19158-PA pbar\|PB21071-PA sinv\|SINV15313-PA cflo\|Cflo_N_g13413t1 hsal\|HSAL18575-PA cbir\|EZA57211.1 lhum\|LH17202-PA acep\|ACEP18121-PA |
| **ant_ortho_group31:** | aech\|AECH19183-PA sinv\|SINV11416-PA pbar\|PB21177-PA cbir\|EZA60641.1 cflo\|Cflo_N_g14753t1 lhum\|LH20531-PA hsal\|HSAL25291-PA acep\|ACEP12023-PA |
| **ant_ortho_group32:** | aech\|AECH22662-PA sinv\|SINV16923-PA pbar\|PB20982-PA cbir\|EZA57116.1 cflo\|Cflo_N_g8778t1 hsal\|HSAL16402-PA lhum\|LH17274-PA acep\|ACEP25594-PA |
| **ant_ortho_group33:** | aech\|AECH24719-PA lhum\|LH12874-PA hsal\|HSAL20623-PA cflo\|Cflo_N_g4134t1 pbar\|PB16581-PA cbir\|EZA51642.1 sinv\|SINV16832-PA acep\|ACEP23729-PA |
| **ant_ortho_group34:** | aech\|AECH15710-PA sinv\|SINV15275-PA pbar\|PB21087-PA lhum\|LH12090-PA cbir\|EZA56748.1 hsal\|HSAL13314-PA cflo\|Cflo_N_g6159t1 acep\|ACEP12105-PA |
| **ant_ortho_group35:** | aech\|AECH14803-PA lhum\|LH19674-PA cbir\|EZA46885.1 pbar\|PB26285-PA sinv\|SINV20040-PA cflo\|Cflo_N_g9310t1 hsal\|HSAL10731-PA acep\|ACEP23915-PA |
| **ant_ortho_group36:** | aech\|AECH22464-PA pbar\|PB18152-PA cflo\|Cflo_N_g2067t1 cbir\|EZA49586.1 sinv\|SINV13896-PA hsal\|HSAL21618-PA acep\|ACEP15728-PA lhum\|LH15947-PA |
| **ant_ortho_group37:** | aech\|AECH13559-PA sinv\|SINV13615-PA lhum\|LH22943-PA cflo\|Cflo_N_g805t1 hsal\|HSAL23212-PA cbir\|EZA52974.1 acep\|ACEP11741-PA pbar\|PB26069-PA |
| **ant_ortho_group38:** | aech\|AECH23179-PA sinv\|SINV24678-PA cflo\|Cflo_N_g5843t1 pbar\|PB20858-PA lhum\|LH21042-PA cbir\|EZA57645.1 hsal\|HSAL16835-PA acep\|ACEP21736-PA |
| **ant_ortho_group39:** | aech\|AECH12186-PA sinv\|SINV18581-PA pbar\|PB24416-PA cbir\|EZA51952.1 lhum\|LH24749-PA cflo\|Cflo_N_g7218t1 hsal\|HSAL10319-PA acep\|ACEP18030-PA |
| **ant_ortho_group40:** | aech\|AECH16769-PA sinv\|SINV11407-PA cflo\|Cflo_N_g7793t1 cbir\|EZA61286.1 lhum\|LH23203-PA pbar\|PB21149-PA hsal\|HSAL18453-PA acep\|ACEP17100-PA |
| **ant_ortho_group41:** | aech\|AECH16380-PA cflo\|Cflo_N_g11945t1 sinv\|SINV20237-PA lhum\|LH18599-PA pbar\|PB14855-PA hsal\|HSAL10904-PA cbir\|EZA61966.1 acep\|ACEP20171-PA |
| **ant_ortho_group42:** | aech\|AECH21669-PA sinv\|SINV18265-PA pbar\|PB25325-PA lhum\|LH18100-PA cflo\|Cflo_N_g2498t1 hsal\|HSAL22485-PA cbir\|EZA48741.1 acep\|ACEP22785-PA |
| **ant_ortho_group43:** | aech\|AECH20463-PA cflo\|Cflo_N_g12635t1 sinv\|SINV14733-PA cbir\|EZA61629.1 hsal\|HSAL20358-PA lhum\|LH15282-PA pbar\|PB23134-PA acep\|ACEP17667-PA |
| **ant_ortho_group44:** | aech\|AECH16054-PA sinv\|SINV26155-PA cflo\|Cflo_N_g1345t1 hsal\|HSAL16521-PA lhum\|LH24594-PA pbar\|PB21645-PA cbir\|EZA62123.1 acep\|ACEP12658-PA |
| **ant_ortho_group45:** | aech\|AECH16055-PA sinv\|SINV26162-PA cflo\|Cflo_N_g1347t1 pbar\|PB26941-PA hsal\|HSAL16522-PA acep\|ACEP12661-PA cbir\|EZA62124.1 lhum\|LH24596-PA |
| **ant_ortho_group46:** | aech\|AECH16102-PA cflo\|Cflo_N_g9087t1 sinv\|SINV10313-PA hsal\|HSAL22502-PA pbar\|PB23609-PA lhum\|LH16879-PA acep\|ACEP12611-PA cbir\|EZA51587.1 |
| **ant_ortho_group47:** | aech\|AECH14327-PA sinv\|SINV20073-PA lhum\|LH12980-PA cbir\|EZA60936.1 cflo\|Cflo_N_g1853t1 pbar\|PB14287-PA hsal\|HSAL18248-PA acep\|ACEP18884-PA |
| **ant_ortho_group48:** | aech\|AECH16143-PA pbar\|PB21415-PA sinv\|SINV18058-PA cflo\|Cflo_N_g13230t1 lhum\|LH11418-PA hsal\|HSAL14391-PA cbir\|EZA51197.1 acep\|ACEP18368-PA |
| **ant_ortho_group49:** | aech\|AECH20086-PA pbar\|PB12145-PA cflo\|Cflo_N_g3297t1 cbir\|EZA51115.1 hsal\|HSAL16245-PA sinv\|SINV14837-PA lhum\|LH23550-PA acep\|ACEP24568-PA |
| **ant_ortho_group50:** | acep\|ACEP12787-PA aech\|AECH23266-PA sinv\|SINV10867-PA cbir\|EZA53619.1 lhum\|LH23770-PA cflo\|Cflo_N_g6471t1 hsal\|HSAL14336-PA pbar\|PB15686-PA |
| **ant_ortho_group51:** | aech\|AECH11298-PA sinv\|SINV10955-PA pbar\|PB24857-PA lhum\|LH17650-PA cflo\|Cflo_N_g12573t1 cbir\|EZA60397.1 hsal\|HSAL20965-PA acep\|ACEP14297-PA |
| **ant_ortho_group52:** | aech\|AECH21954-PA cflo\|Cflo_N_g14472t1 pbar\|PB15278-PA sinv\|SINV20653-PA cbir\|EZA49042.1 lhum\|LH16616-PA hsal\|HSAL17254-PA acep\|ACEP19118-PA |
| **ant_ortho_group53:** | acep\|ACEP12864-PA aech\|AECH20607-PA sinv\|SINV17328-PA pbar\|PB25640-PA cflo\|Cflo_N_g169t1 lhum\|LH23731-PA cbir\|EZA47005.1 hsal\|HSAL21419-PA |
| **ant_ortho_group54:** | aech\|AECH22415-PA pbar\|PB13074-PA sinv\|SINV10209-PA cflo\|Cflo_N_g3488t1 lhum\|LH21061-PA cbir\|EZA53130.1 hsal\|HSAL14086-PA acep\|ACEP25027-PA |
| **ant_ortho_group55:** | aech\|AECH20821-PA sinv\|SINV14333-PA pbar\|PB25564-PA hsal\|HSAL15885-PA lhum\|LH15454-PA cflo\|Cflo_N_g4477t1 cbir\|EZA56654.1 acep\|ACEP24479-PA |
| **ant_ortho_group56:** | aech\|AECH15853-PA cflo\|Cflo_N_g3114t1 cbir\|EZA57266.1 lhum\|LH17899-PA sinv\|SINV11984-PA pbar\|PB24208-PA hsal\|HSAL11279-PA acep\|ACEP11016-PA |
| **ant_ortho_group57:** | aech\|AECH16016-PA pbar\|PB20482-PA cflo\|Cflo_N_g14448t1 hsal\|HSAL14162-PA lhum\|LH13309-PA sinv\|SINV24478-PA acep\|ACEP14724-PA cbir\|EZA47733.1 |
| **ant_ortho_group58:** | aech\|AECH19020-PA cbir\|EZA52611.1 lhum\|LH21865-PA pbar\|PB13598-PA hsal\|HSAL14793-PA cflo\|Cflo_N_g13888t1 acep\|ACEP20945-PA sinv\|SINV10540-PA |
| **ant_ortho_group59:** | aech\|AECH14089-PA sinv\|SINV22903-PA pbar\|PB22277-PA lhum\|LH11880-PA cflo\|Cflo_N_g5388t1 cbir\|EZA58356.1 hsal\|HSAL10018-PA acep\|ACEP27268-PA |
| **ant_ortho_group60:** | aech\|AECH14890-PA sinv\|SINV17083-PA cflo\|Cflo_N_g12037t1 pbar\|PB12486-PA lhum\|LH14928-PA cbir\|EZA50219.1 hsal\|HSAL21543-PA acep\|ACEP17847-PA |
| **ant_ortho_group61:** | aech\|AECH18015-PA pbar\|PB25139-PA sinv\|SINV18710-PA lhum\|LH24054-PA cbir\|EZA49082.1 cflo\|Cflo_N_g7315t1 hsal\|HSAL22074-PA acep\|ACEP25409-PA |
| **ant_ortho_group62:** | aech\|AECH18016-PA sinv\|SINV18730-PA lhum\|LH24050-PA pbar\|PB25140-PA cbir\|EZA49079.1 cflo\|Cflo_N_g9930t1 hsal\|HSAL10493-PA acep\|ACEP25411-PA |
| **ant_ortho_group63:** | acep\|ACEP13091-PA sinv\|SINV24065-PA cbir\|EZA56003.1 lhum\|LH13085-PA pbar\|PB14400-PA cflo\|Cflo_N_g6521t1 hsal\|HSAL10186-PA aech\|AECH11524-PA |
| **ant_ortho_group64:** | aech\|AECH16667-PA pbar\|PB19070-PA lhum\|LH15640-PA cflo\|Cflo_N_g7594t1 hsal\|HSAL23774-PA cbir\|EZA57377.1 acep\|ACEP11219-PA sinv\|SINV24273-PA |
| **ant_ortho_group65:** | aech\|AECH18415-PA cflo\|Cflo_N_g8976t1 hsal\|HSAL21999-PA sinv\|SINV19435-PA lhum\|LH16526-PA cbir\|EZA54963.1 pbar\|PB22883-PA acep\|ACEP10522-PA |
| **ant_ortho_group66:** | aech\|AECH23470-PA pbar\|PB20674-PA sinv\|SINV16932-PA cflo\|Cflo_N_g8789t1 lhum\|LH20676-PA cbir\|EZA57100.1 hsal\|HSAL16382-PA acep\|ACEP25615-PA |
| **ant_ortho_group67:** | aech\|AECH27212-PA lhum\|LH18247-PA cflo\|Cflo_N_g5968t1 cbir\|EZA62770.1 pbar\|PB25362-PA hsal\|HSAL16916-PA acep\|ACEP22824-PA sinv\|SINV18244-PA |
| **ant_ortho_group68:** | aech\|AECH21571-PA sinv\|SINV15901-PA pbar\|PB12779-PA cbir\|EZA57436.1 lhum\|LH11780-PA acep\|ACEP15326-PA cflo\|Cflo_N_g14324t1 hsal\|HSAL19554-PA |
| **ant_ortho_group69:** | aech\|AECH21585-PA cflo\|Cflo_N_g2515t1 cbir\|EZA57426.1 sinv\|SINV15900-PA lhum\|LH16583-PA hsal\|HSAL13672-PA pbar\|PB12785-PA acep\|ACEP15314-PA |
| **ant_ortho_group70:** | sinv\|SINV15757-PA pbar\|PB10624-PA hsal\|HSAL17019-PA cflo\|Cflo_N_g13194t1 lhum\|LH22552-PA cbir\|EZA62666.1 acep\|ACEP10879-PA aech\|AECH16404-PA |
| **ant_ortho_group71:** | acep\|ACEP13596-PA sinv\|SINV23613-PA pbar\|PB26426-PA cflo\|Cflo_N_g14984t1 lhum\|LH25694-PA cbir\|EZA51729.1 hsal\|HSAL15760-PA aech\|AECH20706-PA |
| **ant_ortho_group72:** | cflo\|Cflo_N_g1211t1 cbir\|EZA47600.1 pbar\|PB23837-PA sinv\|SINV13084-PA hsal\|HSAL17705-PA lhum\|LH18681-PA acep\|ACEP23981-PA aech\|AECH17423-PA |
| **ant_ortho_group73:** | cflo\|Cflo_N_g2115t1 pbar\|PB20522-PA sinv\|SINV24080-PA cbir\|EZA53890.1 hsal\|HSAL22507-PA lhum\|LH14341-PA acep\|ACEP17000-PA aech\|AECH12429-PA |
| **ant_ortho_group74:** | cflo\|Cflo_N_g3085t1 pbar\|PB19383-PA sinv\|SINV16875-PA cbir\|EZA53065.1 lhum\|LH17799-PA hsal\|HSAL21140-PA acep\|ACEP11375-PA aech\|AECH16908-PA |
| **ant_ortho_group75:** | cflo\|Cflo_N_g4508t1 cbir\|EZA46930.1 pbar\|PB25150-PA hsal\|HSAL19013-PA lhum\|LH24344-PA sinv\|SINV11568-PA acep\|ACEP23628-PA aech\|AECH11931-PA |
| **ant_ortho_group76:** | cflo\|Cflo_N_g7671t1 cbir\|EZA51737.1 hsal\|HSAL22839-PA pbar\|PB17362-PA sinv\|SINV17584-PA lhum\|LH10297-PA acep\|ACEP23469-PA aech\|AECH20716-PA |
| **ant_ortho_group77:** | cflo\|Cflo_N_g8614t1 lhum\|LH13374-PA pbar\|PB12902-PA sinv\|SINV18850-PA hsal\|HSAL18874-PA cbir\|EZA46805.1 acep\|ACEP21932-PA aech\|AECH12904-PA |
| **ant_ortho_group78:** | cflo\|Cflo_N_g8619t1 pbar\|PB12895-PA sinv\|SINV18911-PA hsal\|HSAL18876-PA cbir\|EZA49940.1 lhum\|LH21616-PA acep\|ACEP21937-PA aech\|AECH12896-PA |
| **ant_ortho_group79:** | cflo\|Cflo_N_g9753t1 pbar\|PB10337-PA sinv\|SINV10517-PA lhum\|LH20544-PA hsal\|HSAL15083-PA cbir\|EZA55353.1 acep\|ACEP20742-PA aech\|AECH10186-PA |
