# Supplementary material for: Scrutinizing the immune defence inventory of Camponotus floridanus applying total transcriptome sequencing
Source: BMC Genomics. 2015 Jul 22;16(1):540. doi: 10.1186/s12864-015-1748-1 (PMC4508827; doi:10.1186/s12864-015-1748-1)
Supplement: Additional file 20: Table S16. — List of oligonucleotides used for validation of changes of gene expression after immune challenge by qRT-PCR. [file 12864_2015_1748_MOESM20_ESM.docx]

**Additional File 20: Table S16:** List of oligonucleotides used for validation of changes of gene expression after immune challenge by qRT-PCR.

| **Gene name** | **Accession No.** | **Primer name** | **Sequence 5‘-3‘** |
| --- | --- | --- | --- |
| **Housekeeping genes:** |  |  |  |
| *60S ribosomal protein L32* | EFN68969 | Cfl_rpL32_rtF1  Cfl_rpL32_rtR1 | GCCAACAGGCTTCCGTAAAG  TGAGCACGTTCGACGATAGC |
| **Recognition:** |  |  |  |
| *Peptidoglycan-recognition*  *protein-LB* (*PGRP-LB*) | EFN73971 | Cfl_PGRP-LB_rtF2  Cfl_PGRP-LB_rtR2 | ACAGCATGAATTTCGTGAATGC  GCCATGATGGATCACCACATA |
| *PGRP-2* | EFN70060 | Cfl_PGRP-2_rtF2  Cfl_PGRP-2_rtR2 | TGAGGGATGTGGATGGACAC  TTAATTGATGCGCAGCGTTC |
| *Scavenger receptor class B member 1* (*scav*) | EFN70524 | Cfl_scav_rtF1  Cfl_scav_rtR1 | ATGGCTTGAATCCTCGACAG  CAAGAAACGGTACGCCAATC |
| *Alpha-2-macroglobulin-like protein 1 (TEP1)* | EFN69033 | Cfl_TEP1_cdsF1  Cfl_TEP1_cdsR1 | ATATGGAGGCGTCATTTTCG  CCTTCGACACCATCAGGA |
| *CD109 antigen (TEP2)* | EFN73645 | Cfl_TEP2_cdsF1  Cfl_TEP2_cdsR1 | ACGGTGGTATTGGCAGAC  CTTGTGCAGGAATCCAAATG |
| *CD109 antigen (TEP3)* | EFN68790 | Cfl_TEP3_cdsF2  Cfl_TEP3_cdsR2 | TTACCGAGTACGGCAAATCC  AGAAACCTCCGCAAAGTCAA |
| **Signaling and immune defense:** |  |  |  |
| *Relish* (*Rel*) | EFN61437 | Cfl_Rel_rtF1  Cfl_Rel_rtR1 | CACCTTTGCAATTAGCTGCTG  ACCTCCTTCGACTGCGATATG |
| *Receptor-interacting serine/threonine-protein kinase 1 (IMDK1)* | EFN61166 | Cfl_ImdK1_cdsF1  Cfl_ImdK1_cdsR1 | ATGACGACGTTACGATGCAA  TATACTCTTTCGACTCGCTC |
| *Suppressor of cytokine signaling*  *2* (*SOCS2*) | EFN67156 | Cfl_SOCS2_rtF1  Cfl_SOCS2_rtR1 | TTTGTCAGTGTCACGGCAAG  TCGGCAGTCACTCATGTTCA |
| *Parathyroid hormone-related*  *peptide receptor* (*PHR*) | EFN73318 | Cfl_PHR_rtF1  Cfl_PHR_rtR1 | GGTTCACGACCATCAACACA  TGATCTTGGAACATGCAAGG |
| *NF-kappa-B inhibitor cactus 1* | EFN66753 | Cfl_cact1_rtF1  Cfl_cact1_rtR1 | AATAAGCTGACGCTCGGACA  AACGCAACAGAAGTCGCACT |
| *Mitogen-activated protein kinase kinase kinase 7 (MAPKKK7)* | EFN63041 | Cfl_MAPKKK7_cdsF1  Cfl_MAPKKK7_cdsR1 | TGGAGTCGTCTGGAAGGG  GGATGGGCAACTCTGGAT |
| *Sushi, von Willebrand factor type A* | EFN63579 | Cfl_sushi_rtF1  Cfl_sushi_rtR1 | TATCTGGAGCGGACACCAAC  TGAATCCATTTTGGCAGGAG |
| *Embryonic polarity protein dorsal* | EFN68841 | Cfl_Dorsal_cdsF1  Cfl_Dorsal_cdsR1 | GTTACAATGGTCGCGCTAT  ACAAACACCTTGCTTGCACA |
| *Hymenoptaecin* (*hym*) | HS410972 | Cfl_HymRT_F5  Cfl_HymRT_R5 | CACAGTAGAAACGAAAACATTCC  ATGAAGTTTCCTGGGCACTCG |
| *Defensin-1* (*def-1*) | HS410966 | Cfl_Def1_rtF1  Cfl_Def1_rtR1 | CGGTAGAGTCTCCGGACTTTT  CGCTATGATTAACACCGAAGC |
| *Tyrosine Hydroxylase* (*TyrOH*) | HS410957 | Cfl_TyHRT_F2  Cfl_TyHRT_R2 | GCCCAGAAGAACCGTGAAAT  TGTTTGCTTGACGACCAATGTC |
| *Probable phenoloxidase subunit*  *CG8193* (*POsub*) | EFN74080 | Cfl_POsub_rtF2  Cfl_POsub_rtR2 | CATAATCTCGGTCACGTTGC  GACGAAAGCGTGGAATCTGT |
| *NADPH--cytochrome P450 reductase nitric oxide synthase 1 (NOS1)* | EFN67037 | Cfl_NOS1_cdsF1  Cfl_NOS1_cdsR1 | CCTTCCGATCCACCTTCT  CTGAAAGGCGCTAGACCA |
| *Metalloproteinase inhibitor*  *3*(*MPI*) | EFN65977 | Cfl_MPI_rtF1  Cfl_MPI_rtR1 | ATAAGGTGGGCCGATACCAC  TTTCCGCCACTGGACTCTAG |
| *Esterase FE4* (*ester*) | EFN65474 | Cfl_ester_rtF1  Cfl_ester_rtR1 | CTTCGATGGGTCAAGAGGAA  TGGAACAATCCCTTGGACAT |
| **Stress-response:** |  |  |  |
| *Transferrin* (*transf*) | EFN62546 | Cfl_transf_rtF1  Cfl_transf_rtR1 | ATTCCAAGCACCCATCAATG  TATAAGGTGCTCGCGGCTAA |
| *Cytochrome P450 18a1* | EFN74585 | Cfl_cP45018a1_rtF2  Cfl_cP45018a1_rtR2 | TACCCAAATAGTGCCGCTGT  CTCCGAACGGCATGAAGTAT |
| **Digestion and storage:** |  |  |  |
| *Chymotrypsin-1* | EFN68337 | Cfl_chymo_rtF1  Cfl_chymo_rtR1 | CTCAACCGTCAAGGAATTGG  ACATTGGCGTAGCAATCTGG |
| *Hexamerin (hex)* | EFN73934 | Cfl_hex_rtF1  Cfl_hex_rtR1 | TGATCAACAACGCTGTGTCC  TTGACGTTCTTGTCGCTGTG |
| *Lipase member H-A* (*lip*) | EFN63276 | Cfl_lip_rtF1  Cfl_lip_rtR1 | ACGCCTTTATTCAGGGCAAG  AAAGTATTCCGCTGCCCTGT |
| **Lysosomal system, Autophagy:** |  |  |  |
| *Lysozyme i-type* (*i-type lyso*) | EFN71839 | Cfl_2Lyso_rtF3  Cfl_2Lyso_rtR3 | GCTCAATCACCGTCCAATGA  GCCCAGTAGCCCCATGTTAT |
| *Lysosomal aspartic protease* | EFN61281 | Cfl_LAP61281_rtF1  Cfl_LAP61281_rtR1 | GATGGAGTGACGCCTGTCTT  GGATCGGAACCACCCAATAT |
| **Wound closure:** |  |  |  |
| *Protein yellow (Major royal jelly protein)* | EFN61809 | Cfl_yellow_rtF1  Cfl_yellow_rtR1 | GAAACCCGATAATCGCACCT  AGCTTGCCAGTGTCGAGAAC |
| *Acidic mammalian chitinase (chitolectin)* | EFN71329 | Cfl_chito_rtF1  Cfl_chito_rtR1 | TGATCGTCGGTGTACCATCC  TCATTGTAGCCCAGCATTCC |
| **Other function:** |  |  |  |
| *Zinc carboxypeptidase A 1* (*zcp*) | EFN74038 | Cfl_zcp_rtF1  Cfl_zcp_rtR1 | ACGCGAGATCAAAGGTGTCA  TGCTGGTCAGCACTTGATGT |
| *Vitellogenin* | EFN64902 | Cfl_vitel_rtF1  Cfl_vitel_rtR1 | CTGAACACTTCCATGCCATGT  CCGCCTCTATGTCGCTAACA |
| **Hypothetical proteins:** |  |  |  |
| *hypothetical protein* (*hp67112*) | EFN67112 | Cfl_hp67112_rtF1  Cfl_hp67112_rtR1 | GGCAGTTTGCAGGAAATACG  AAATTACACCGTTGCCTTCG |
| *hypothetical protein (chitin-binding)* | EFN72799 | Cfl_hpchit_rtF1  Cfl_hpchit_rtR1 | TCCGCAAGCAAAGATAGTCAG  AAAACCAGGGGACACACAATC |
| *hypothetical protein (Pacifastin inhibitor)* | EFN62229 | Cfl_hppaci_rtF1  Cfl_hppaci_rtR1 | TTCTGCTGCACAGGAGGAAC  GGTGGAATCGTCACTGATGG |
| *hypothetical protein* | EFN70940 | Cfl_hp70940_rtF1  Cfl_hp70940_rtR1 | GCAACTGGTAGATCGTGCAA  GTCCAACAGCCCTGGATAGA |
