# Supplementary material for: Scrutinizing the immune defence inventory of Camponotus floridanus applying total transcriptome sequencing
Source: BMC Genomics. 2015 Jul 22;16(1):540. doi: 10.1186/s12864-015-1748-1 (PMC4508827; doi:10.1186/s12864-015-1748-1)
Supplement: Additional file 22: Table S18. — Accession numbers of the sequentially used query sequences from different insects. [file 12864_2015_1748_MOESM22_ESM.docx]

**Additional File 22: Table S18:** Accession number of the sequentially used query sequences from different insects.

| **Immune effectors** | **Query sequences (GenBank accession no.)** |
| --- | --- |
| Chitinase | *Manduca sexta* (AAB53952.1), *Anopheles gambiae* (AAB87764.1), *Aedes aegypti* (AAB81849.1), *Phaedon cochleariae* (CAA77014.1) and *Bombyx mori* (BAC67246.1) |
| Lysozymes | *Drosophila melanogaster* (NP523882, NP476827, NP476823, NP524869, NP476829, NP523881, NP476828), *Anopheles gambiae* (AAC47326), *Bombyx mori* (AAB40947) and *Manduca sexta* (AAB31190) |
| Prophenoloxidase | *Drosophila melanogaster* (NP_610443.1), *Apis mellifera* (AAO72539.2), *Aedes aegypti* (XP_001663691.1) and *Anopheles gambiae* (EAA03423.1) |
| Nitric oxide synthase | *Drosophila melanogaster* (NP_523541.2), *Apis mellifera* (NP_001012980.1), *Anopheles gambiae* (XP_317213.1) and *Manduca sexta* (AAC61262.1) |
| Glutathione S-transferase | *Drosophila melanogaster* (NP_524326.1, NP_611323.1, NP_649894.1, NP_648237.1, NP_610509.2, NP_725653.1), *Apis mellifera* (NP_001171499.1, XP_394562.1, XP_624501.1, XP_624692.1, XP_624662.2), *Anopheles gambiae* (XP_313050.3, AAL59658.1, XP_312009.2, AAP13482.1, XP_311299.1, AAM53611.1) and *Bombyx mori* (NP_001037183.1, NP_001037197.1, NP_001037418.1, NP_001040131.1, NP_001108463.1, NP_001037077.1) |
